# Transcriptomic and immunophenotypic profiling reveals molecular and immunological hallmarks of colorectal cancer tumourigenesis

**DOI:** 10.1136/gutjnl-2022-327608

**Published:** 2022-11-28

**Authors:** Jessica Roelands, Manon van der Ploeg, Marieke E Ijsselsteijn, Hao Dang, Jurjen J Boonstra, James C H Hardwick, Lukas J A C Hawinkels, Hans Morreau, Noel F C C de Miranda

**Affiliations:** 1 Department of Pathology, Leiden University Medical Center, Leiden, The Netherlands; 2 Department of Gastroenterology and Hepatology, Leiden University Medical Center, Leiden, The Netherlands

**Keywords:** colorectal neogenesis, immunogenetics, gene expression

## Abstract

**Objective:**

Biological insights into the stepwise development and progression of colorectal cancer (CRC) are imperative to develop tailored approaches for early detection and optimal clinical management of this disease. Here, we aimed to dissect the transcriptional and immunologic alterations that accompany malignant transformation in CRC and to identify clinically relevant biomarkers through spatial profiling of pT1 CRC samples.

**Design:**

We employed digital spatial profiling (GeoMx) on eight pT1 CRCs to study gene expression in the epithelial and stromal segments across regions of distinct histology, including normal mucosa, low-grade and high-grade dysplasia and cancer. Consecutive histology sections were profiled by imaging mass cytometry to reveal immune contextures. Finally, publicly available single-cell RNA-sequencing data was analysed to determine the cellular origin of relevant transcripts.

**Results:**

Comparison of gene expression between regions within pT1 CRC samples identified differentially expressed genes in the epithelium (n=1394 genes) and the stromal segments (n=1145 genes) across distinct histologies. Pathway analysis identified an early onset of inflammatory responses during malignant transformation, typified by upregulation of gene signatures such as innate immune sensing. We detected increased infiltration of myeloid cells and a shift in macrophage populations from pro-inflammatory HLA-DR^+^CD204^−^ macrophages to HLA-DR^−^CD204^+^ immune-suppressive subsets from normal tissue through dysplasia to cancer, accompanied by the upregulation of the CD47/SIRPα ‘don’t eat me signal’.

**Conclusion:**

Spatial profiling revealed the molecular and immunological landscape of CRC tumourigenesis at early disease stage. We identified biomarkers with strong association with disease progression as well as targetable immune processes that are exploitable in a clinical setting.

**WHAT IS ALREADY KNOWN ON THIS TOPIC**

- Colorectal cancers (CRCs) arise as a consequence of the gradual accumulation of (epi-) genetic alterations in epithelial cells, ultimately leading to uncontrolled proliferation and malignant transformation.
- Our understanding of the biological processes that underlie CRC onset and progression is still insufficient for optimal management of this disease.
- To date, studies that clarify the roles played by different cells of the tumour microenvironment during malignant transformation are still scarce.
- Technologies that profile gene expression while preserving tissue contexture allow the comprehensive investigation of the transcriptional landscape in specific locations within a tissue to gain in-depth insights into biological processes associated with malignant transformation.

**WHAT THIS STUDY ADDS**

- MUC4, IFITM1 and CD81 are specifically deregulated in the epithelium during early-stages of malignant transformation in CRC, while NOTCH3, PDGFRB, Thy1 and Hsp47 are found deregulated in the stroma.
- CRC onset is accompanied by an early activation of innate immune responses and nucleic acid sensing.
- The immune cell composition in pT1 CRC tissues gradually changes from normal tissue, through areas of dysplasia, to cancer.
- The stepwise process of CRC tumourigenesis is accompanied by a shift from pro-inflammatory to immune-suppressive macrophage populations, which specifically express *SIRPA*, and by the expression of CD47(SIRPα-ligand) on tumour cells.

**HOW THIS STUDY MIGHT AFFECT RESEARCH, PRACTICE OR POLICY**

- In this work, we identified proteins associated with CRC tumourigenesis that are promising candidate biomarkers for early detection and adequate staging of CRC.
- Our multiregion transcriptomics approach elucidates biological processes that are at the basis of CRC onset and progression, such as innate immune sensing, that could be targeted for early intervention or prevention.
- The CD47-SIRPα axis might constitute an attractive immunotherapeutic target in CRC.

## Introduction

The paradigm of the adenoma–carcinoma sequence in colorectal cancer (CRC) development has typically been centred on cancer cells^1^; however, it is now clear that cancer progression involves heterologous interactions between transformed cells and their surrounding microenvironment.^2^ Most studies, so far, relied on bulk profiling in advanced stages of CRC to identify cancer driver genes and define biological processes related to cancer.^3–5^ Large-scale genomic and transcriptomic studies have been instrumental for bridging CRC genomics, including microsatellite instability, neoantigen load and other immune-related genomic aberrations to immune-cell infiltration and prognosis in CRC.^5–7^

Different strategies have been applied to define transcriptomic and epigenetic alterations that accompany tumourigenesis,^8–10^ either by comparing normal, adenoma and carcinoma tissues^8 10^ or microdissection of CRC lesions.^9^ More recently, single cell transcriptomics has provided a more granular view of the tumour immune microenvironment through the identification of distinct subsets of stromal, immune and malignant cells and multicellular interaction networks in CRC.^11^ Recent efforts have identified premalignant gene expression programmes by single cell sequencing of precancerous polyps.^12 13^ In addition, the pronounced changes to the tumour microenvironment were observed during malignant transformation, including large shifts in fibroblast subpopulations, enrichment of regulatory T cells and reduced B cells in polyps.^13^

A common limitation of bulk and single-cell approaches is the lack of information on spatial context and cellular composition across distinct regions. Spatial gene expression profiling allows the resolving of biological processes that accompany morphological changes in a tissue.^14 15^ Early-staged CRC (eg, pT1 CRC) is particularly suitable for analyses by spatial methodologies as different histologies are present within the same lesion including healthy tissue, different degrees of dysplasia and cancer, thereby representing the early processes of malignant transformation. This is a unique model to elucidate the biological processes that support CRC tumourigenesis as those histologies share a common origin with identical (germline) genetic background and comparable exposure to environmental factors such as the microbiome.

We mapped the transcriptional changes occurring during CRC onset by applying digital spatial profiling (DSP) on matched normal mucosa, low-grade and high-grade dysplasia and cancer in pT1 CRC lesions. The parallel analysis of epithelial and stromal fractions allowed the dissection of biological alterations occurring in tumour cells, in stromal cells or on both compartments of the tumour microenvironment. Genes and pathways that were consistently deregulated during CRC progression were identified. Furthermore, we detected marked changes in the composition and functional orientation of the immune microenvironment during CRC onset with a prominent role of the innate immune system. In particular, we provide evidence for a shift in macrophage populations during the stepwise progression from normal tissue to CRC, accompanied by the upregulation of the CD47/SIRPα axis.

## Materials and methods

### Human samples

Patient samples were obtained by endoscopic submucosal dissection at the Department of Gastroenterology of the Leiden University Medical Center. Patients did not receive any treatment prior to the endoscopic procedure. Tissues were formalin-fixed and paraffin embedded (FFPE).

### Patient involvement

While early detection and treatment are imperative to a patient’s outcome, the clinical management of patients diagnosed with early-stage cancer can be further improved. The current study advances our understanding of the biological mechanisms underlying the onset of CRC as a first, but essential step, to identify biomarkers that support the tailored treatment of patients with early-staged CRC. We plan to communicate the results of this study through social media and lectures co-organised with patient advocacy groups. In collaboration with Fight Colorectal Cancer (https://fightcolorectalcancer.org/crc-research/advisory-committees/our-medical-experts/), we aim to ensure that patients with CRC are well-informed about developments in this field.

### In situ hybridisation and digital spatial profiling

To prepare slides for DSP, 5 µm thick FFPE sections of eight different patients (n=8) were deparaffinised, heated in ER2 solution (Leica) at 100°C for 20 min and treated with 1 µg/mL proteinase K at 37°C for 15 min. An overnight in situ hybridisation was performed as described with a final probe concentration of 4 nM per probe. Slides were washed twice at 37°C for 25 min with 50% formamide/2X SSC buffer to remove unbound probes.

Prepared slides were incubated with immunofluorescent antibodies and GeoMx Cancer Transcriptome Atlas (CTA, V.2.0) profiling reagents simultaneously. Pan-cytokeratin (PanCK; AE1+AE3, Novus Biologicals, Cat# NBP2-33200AF488) was used for identification of epithelial cells, Vimentin (E-5 clone from Santa Cruz, Cat# sc-373717) for stromal cells, CD45 (D9M8I from CST, Cat# 13917. internally conjugated) for all haematopoietic cells and DNA GeoMx Nuclear Stain (Cat# 121303303) for nuclei. Stained slides were loaded onto the GeoMx instrument and scanned.

For each ESD, nine geometrical regions of interest (ROIs) with different histologies were selected by a pathologist. We have used geometrical shapes, including rectangular shapes and polygons, to specifically select regions based on histology, including normal tissue, transition, low-grade and high-grade dysplasia and cancer. The resulting surface areas and number of nuclei for each ROI can vary depending on the respective size of the distinct regions and the cellular density in those (online supplemental table S1). Each ROI was UV-illuminated twice, once for the PanCK segment and once for the Vimentin segment. The resulting areas of illumination (AOIs, n=144) had a surface area in the range of 15 000–350 000 µm^2^, encompassing between 102 and 5054 nuclei.

10.1136/gutjnl-2022-327608.supp2 Supplementary data

### DSP data processing and normalisation

Raw data was available in the GeoMx DSP Analysis Suite. We performed the segmental and biological probe quality control (QC) following NanoString’s Cancer Transcriptome Atlas Normalization guidelines. AOI QC was performed in the DSP Analysis Suite to flag low-performing AOIs using the following settings: raw reads threshold <1000 reads, per cent aligned reads <80%, sequencing saturation <50%, negative probe count geomean <10, no template PCR control count >60, minimum nuclei <100 and minimum surface area of 600 µm². A total of 31 AOIs were flagged with a low negative probe count and one sample with a low sequencing saturation. Probe QC was performed with default settings excluding probes from target count calculation in all segments if (geomean probe in all segments)/(geomean probes within target) ≤ 0.1 and if fails Grubbs’ outlier test in ≥20% of the segments. If a probe fails Grubb’s outlier test in a specific segment, the probe was excluded from target count calculation in that segment. The limit of quantitation was calculated using two SDs of the geomean of the negative probes.

To determine whether flagged AOIs need to be removed from the study, we compared background to on-target signal strength. We performed Quartile 3 count (Q3) normalisation in the DSP Analysis Suite to account for technical effects between AOIs (eg, segment area, amount of targetable messenger RNA, in-situ hybridisation binding). While most AOIs have a Q3 signal substantially higher than the NegProbe count, two AOIs needed to be dropped as in these the Q3 signal was comparable to the background. Following QC, the resulting gene expression matrix consisted of 141 AOIs that passed QC and a total of 1825 genes. Q3-normalised expression data was exported from the DSP Analysis Suite. All downstream analysis was performed in R (V.4.0.3 or later). Dimension-reduction of the expression matrix was performed using t-Distributed Stochastic Neighbor Embedding by R package ‘Rtsne’ (V.0.15) and visualised using ggplot2 (V.3.3.3).

### Data exploration using Spatial Organ Atlas— Colon

Raw counts from the Spatial Organ Atlas were downloaded from the NanoString website (https://nanostring.com/resources/soa-human-colon-minerva-story/).^16^ The Spatial Organ Atlas contains The GeoMx Whole Transcriptome Atlas Human Colon data from four healthy individuals (two men and two women). The raw gene counts matrix from the pT1 samples was merged with the raw counts of the Spatial Organ Atlas. Q3-normalisation was applied to the merged matrix. Boxplots of Log2 transformed, normalised gene expression were generated using ggplot2 in R.

### Gene set enrichment analysis

Single sample gene set enrichment analysis (ssGSEA) was performed on the log2 transformed, normalised gene expression matrix.^17^ Wiki pathways^18^ were retrieved using the downloadPathwayArchive function of the R package ‘rWikiPathways’ (parameters: organism = ‘Homo sapiens’, format=‘gmt’) on 10 May 2021. All pathways with <2 genes available in the GeoMx CTA probeset were removed. The WikiPathways database^18^ was used to extract non-redundant biological pathways suitable for ssGSEA. Downloaded gene sets were tested for applicability for ssGSEA in the CTA expression matrix (1825 genes) by employing the The Cancer Genome Atlas Colon Adenocarcinoma (TCGA-COAD) data set. The ssGSEA was performed on the full TCGA-COAD gene expression matrix and the same matrix filtered to only include the CTA genes. The Pearson correlation between obtained enrichment scores was determined for each gene set. Gene sets with a Pearson correlation coefficient <0.6, we deemed unsuitable for ssGSEA in our GeoMx CTA data set and were excluded for further analysis. In the cases that multiple gene sets describe the same biological process or pathway, only the pathway with the highest correlation was maintained. Pathways that describe a specific disease setting or organ were excluded. This resulted in a list of 117 WikiPathways.

### Immune cell deconvolution

To estimate relative abundancies from the expression data, we implemented Consensus Tumour Microenvironment cell Estimation (ConsensusTME), a method that relies on integrated gene sets from multiple sources and has been optimised for each individual cancer type, using R package ConsensusTME (V.0.0.1.9).^19^ Parameters were set to ‘COAD’ to specify cancer type and ‘ssgsea’ as statistical method. Corroborating the reliability of this method, we observed low enrichment scores of the immune cell subsets in AOIs from the epithelial compartment (online supplemental figure 1).

10.1136/gutjnl-2022-327608.supp7 Supplementary data

### Immunohistochemistry

Details on immunohistochemical detection of selected proteins of interest are available in online supplemental methods and online supplemental table S2.

10.1136/gutjnl-2022-327608.supp1 Supplementary data

10.1136/gutjnl-2022-327608.supp3 Supplementary data

### Imaging mass cytometry data analysis

Details on imaging mass cytometry are available in online supplementary methods and used antibodies are included in online supplemental table S3.

10.1136/gutjnl-2022-327608.supp4 Supplementary data

### Single-cell RNA-sequencing analysis

Details on single-cell RNA-sequencing (scRNA-seq) analysis are available in online supplemental methods.

### Statistical analyses

Differentially expressed genes between PanCK and Vimentin segments were determined using a paired t-test and corresponding false discovery rate (FDR) was calculated by the Benjamini-Hochberg method. Stepwise-differential expression analysis between regions with distinct progressive status was calculated using an unpaired t-test. The Spearman correlation test was used to correlate to define genes/pathways/proteins that are increasing or decreasing during the stepwise transition of normal colon to carcinoma. For this test, the region parameter was treated as an ordinal variable (1=normal; 2=transition area; 3=low-grade dysplasia, 4=high-grade dysplasia, 5=carcinoma). This analysis was performed for the PanCK and the Vimentin segment separately. One-way analysis of variance (ANOVA) was applied to identify differential gene expression between regions.

## Results

### Intratumoural gene expression profiling of CRC tumourigenesis

**Figure 1:**
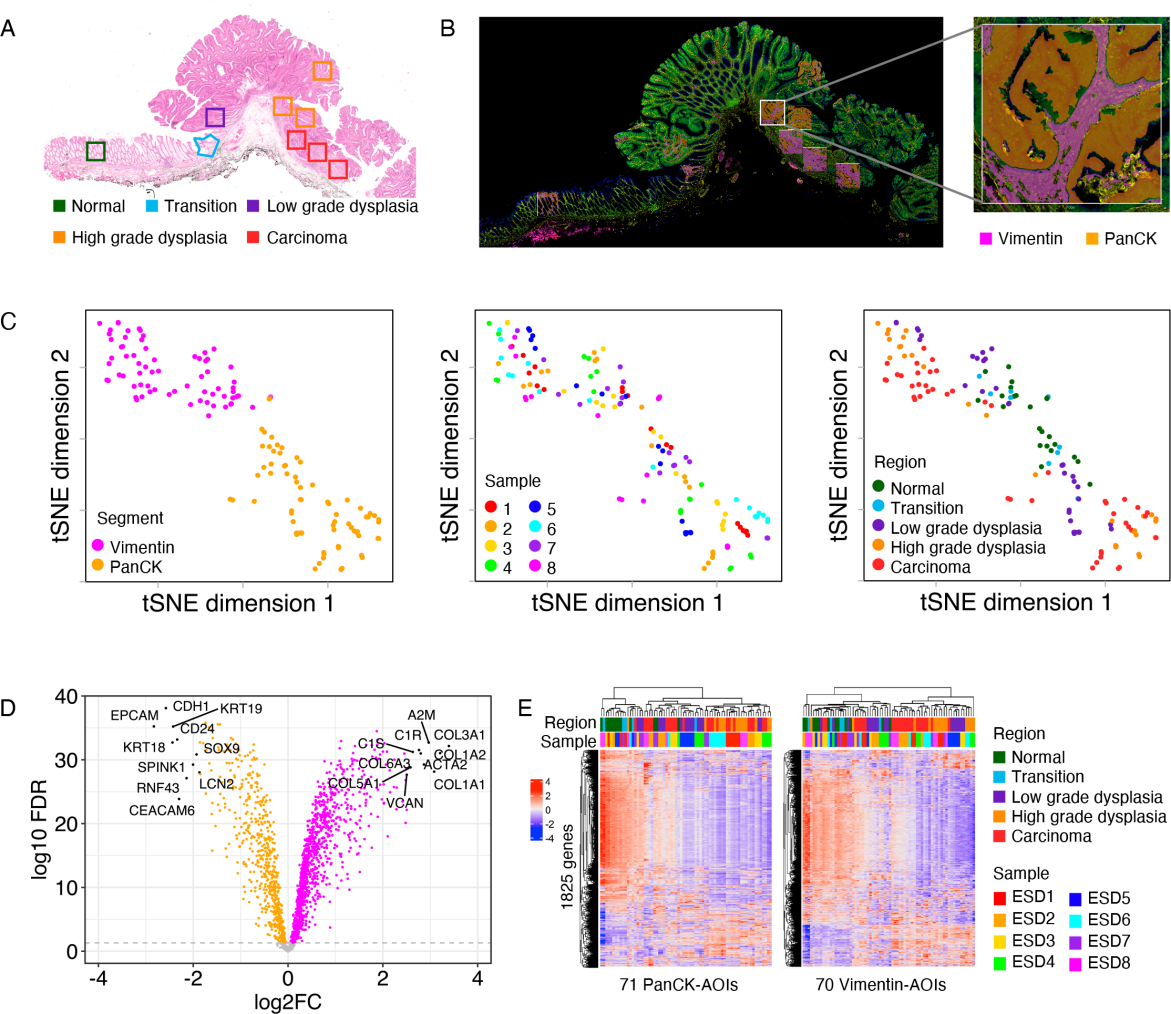
Digital spatial profiling of early-stage CRC. (A) Representative example of a pT1 CRC sample stained by H&E. Selected ROIs with different histological features are annotated. (B) Immunofluorescent detection of PanCK and Vimentin on the same sample as in panel (A). Artificial overlay of tissue segmentation is indicated for each ROI, visualising Vimentin+ (pink) and PanCK+ (orange) segments. Inset: higher magnification of an individual ROI. (C) Dimensionality reduction visualisation of all AOIs according to overall gene expression profiles by tSNE. The tSNE plots are annotated by segment (left), sample ID (middle) and histological region (right). (D) Volcano plot of differentially expressed genes between PanCK and Vimentin segments by paired t-test. FDR is calculated using the Benjamini-Hochberg method. (E) Heatmaps of gene expression (n=1825) using unsupervised clustering for PanCK AOIs (n=71, left) and Vimentin AOIs (n=70, right). Heatmaps are annotated by histological region and sample ID. AOI, areas of illumination; CRC, colorectal cancer; FDR, false discovery rate; PanCK, pan-cytokeratin; ROI, region of interest; tSNE, t-Distributed Stochastic Neighbour Embedding.

To decipher the molecular changes that occur during the onset and progression of CRC, we profiled eight pT1 CRC samples using the GeoMx DSP technology. For each sample, nine ROIs were selected, encompassing normal mucosa, areas of transition between normal mucosa and dysplasia, regions with low-grade and high-grade dysplasia and carcinoma tissue (figure 1A). To specifically examine intratumoural transcriptional alterations within epithelial and stromal compartments, we interrogated, separately, cytokeratin positive (PanCK+) and vimentin positive (Vimentin+) fractions within each ROI (figure 1B).

Data exploration using dimension reduction by t-Distributed Stochastic Neighbour Embedding (tSNE) revealed, as expected, a clear segregation of AOIs by segment (PanCK/Vimentin) (figure 1C, *left panel*). The large majority of genes in the CTA collection was differentially expressed between segments: 583 genes were upregulated in the epithelial segments and 1068 genes were upregulated in the stromal segments (paired t-test; Benjamini-Hochberg method FDR<0.05) (figure 1D). Mapping of AOIs in the tSNE was related to the histological features of the profiled regions (figure 1C). Interestingly, normal and transition areas separated from high-grade dysplasia and carcinoma AOIs within both epithelial and stromal segments. Epithelial segments of low-grade dysplasia were positioned in between these histologies, thereby supporting the validity of our approach to investigate stepwise alterations in CRC. In the PanCK cluster, we observed that AOIs corresponding to high-grade dysplasia and carcinoma formed small subclusters by sample (figure 1C), most likely representing inter-patient variability regarding tumour-associated transcriptomic profiles. Overall, these findings support that CRC onset is not only associated with transcriptional alterations in (pre-) malignant epithelial cells but also in the surrounding stromal cells.

For a high-level overview, we subsequently visualised the expression of all 1825 CTA genes in unsupervised clustered heatmaps, per segment (figure 1E). In total, 242 genes were upregulated during tumourigenesis in the epithelial segment and 288 genes in the stromal compartment (Spearman correlation, FDR<0.05). Interestingly, a larger number of genes decreased in expression during CRC progression (ie, 1152 genes in the epithelium and 857 genes in the stroma, Spearman correlation, FDR<0.05) and those genes were largely consistent across samples, likely reflecting the common loss of physiological features of the tissues during malignant transformation (figure 1E, online supplemental figure 2).

10.1136/gutjnl-2022-327608.supp8 Supplementary data

### Gene expression changes associated to stepwise progression in CRC

**Figure 2:**
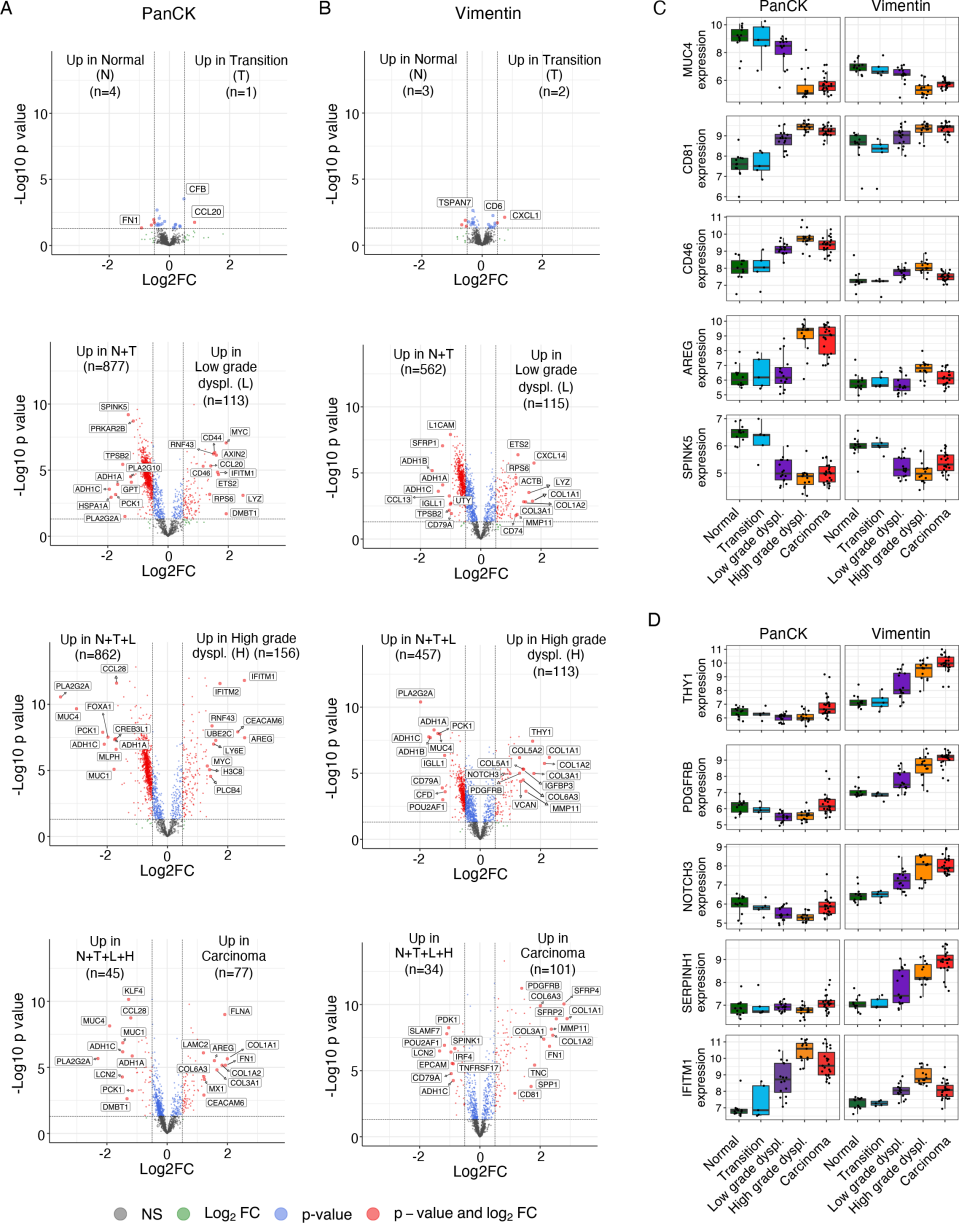
(A–B) Volcano plots showing differentially expressed genes between distinct histologies in stepwise comparisons (unpaired t-test). Reference groups for comparisons are indicated. Analysis was performed for the epithelial segment (PanCK) (A) and stromal segment (Vimentin) (B), separately. A p value of 0.05 (−log10 p value of 1.30103) and log2FC of 0.5 were used as cut-offs. Differentially expressed genes with the highest and lowest log2FC and selected genes of interest are labelled. (C) Boxplots of log2-transformed, normalised gene expression of five differentially expressed genes detected in the epithelium and corresponding gene expression in the stromal segment. (D) Boxplots of five differentially expressed genes identified in the stroma (vimentin) and corresponding gene expression in the epithelial segment. Log2FC, log2 fold change; PanCK, pan-cytokeratin.

To gain insight into gene expression changes associated with the stepwise progression from normal tissue to carcinoma, we assessed differential gene expression between regions with distinct histologies (figure 2A, B). As expected, only small differences were identified between normal mucosa and the transition area between normal mucosa and dysplasia, both for the epithelial and stromal compartments (figure 2A, B, upper panels). Nevertheless, these changes may reflect important initial events of tumourigenesis. Interestingly, complement factor B (*CFB*) and C-C motif chemokine 20 (*CCL20*) were upregulated in the epithelium, while C-X-C Motif Chemokine Ligand 1 (*CXCL1*) was increased in the stroma of transition areas, providing cues for an early onset of inflammatory processes. Moving to areas of dysplasia, substantial differences in gene expression were observed, both in the epithelium as well as stromal compartments (figure 2A, B).

**Figure 3:**
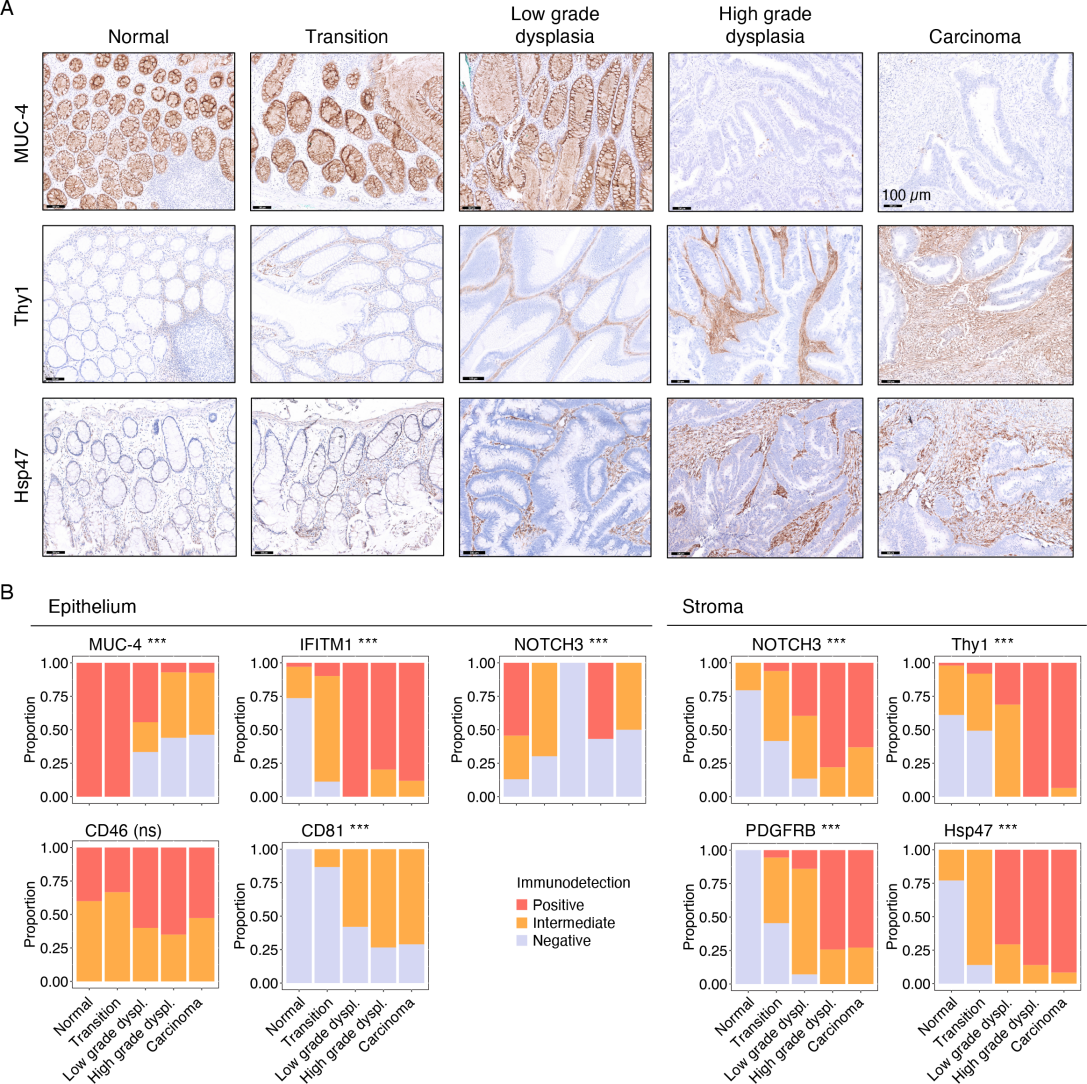
Differential protein abundance of candidate biomarkers in early-stage CRC. (A) Protein abundance of candidate biomarkers in the early-stage CRC samples that were profiled using DSP (n=8). Representative images of IHC detection for MUC-4, Thy1 and Hsp47 across regions with distinct histology (normal, transition, low-grade dysplasia, high-grade dysplasia and carcinoma). (B) Protein abundance of candidate biomarkers in an independent validation set of 20 pT1 CRC samples. Stacked bar chart reflects proportion of samples in each scored category. Significance of Spearman correlation is indicated by asterisks. ***p<0.001. CRC, colorectal cancer; DSP, digital spatial profiling; IHC, immunohistochemistry.

Specific genes demonstrated clear changes in expression between neighbouring regions of distinct histologies across all patients and are therefore promising biomarkers for early disease interception (online supplemental figure 3). For illustrative purposes, we selected some genes that demonstrated high differential expression between regions (ANOVA) (online supplemental figure 4, online supplemental figure 5), and for which commercial antibodies were available for their validation at the protein level. *MUC4*,^20^ and *AREG* 21 (ie, amphiregulin), previously described to be associated to CRC tumourigenesis, and the potentially novel biomarkers *CD81*, *CD46* and *SPINK5* were strongly deregulated in the epithelial segments of the tumours (figure 2C). In the stroma, *THY1*, *PDGFRB*, *NOTCH3*, *SERPINH1* (ie, Hsp47) and *IFITM1* were selected for further analysis (figure 2D). Coordinated gene expression changes across both epithelial and stromal segments were observed for *IFITM1*, *SPINK5* and *CD81*. Changes in expression of all selected genes were largely consistent between patients (online supplemental figure 4). To verify whether these alterations in gene expression translated to changes in protein abundance, immunodetection of the 10 targets was performed on consecutive sections of the same pT1 lesions (figure 3A, online supplemental figure 5) as well as on 20 additional pT1 CRC samples (figure 3B). In the epithelial segment, we confirmed deregulation of MUC-4 (Spearman Rho=−0.68, p=3.96×10^−14^, figure 3A, B), IFITM1 (Spearman Rho=0.73, p=1.59×10^−16^) and CD81 (Spearman Rho=0.45, p=5.43×10^−6^, online supplemental figure 5). In the stromal segment, we confirmed increased protein expression for NOTCH3 (Spearman Rho=0.65, p=4.59×10^−12^), Thy1 (Spearman Rho=0.76, p=5.42×10^−19^), PDGFRB (Spearman Rho=0.81, 2.27×10^−23^) and Hsp47 (Spearman Rho=0.82, p=1.20×10^−23^) during the stepwise progression of CRC across samples (figure 3A, online supplemental figure 5). For CD46, only marginal differences were detected in the epithelium with large variation between samples (Spearman Rho=0.16, p=0.127, online supplemental figure 5). We did not detect any protein expression of Serine Peptidase Inhibitor Kazal Type 5 (*SPINK5*) in colorectal tissues and expression of amphiregulin (*AREG*) could not reliably be assessed (data not shown).

10.1136/gutjnl-2022-327608.supp9 Supplementary data

10.1136/gutjnl-2022-327608.supp10 Supplementary data

10.1136/gutjnl-2022-327608.supp11 Supplementary data

To investigate whether the normal tissue region identified in pT1 samples indeed represented ‘healthy’ colorectal mucosa, we investigated the expression of the genes of interest in normal colon tissue from healthy individuals using the recently published Spatial Organ Atlas from NanoString.^16^ Most of the selected genes that were deregulated from normal epithelium through the stepwise transition to cancer, demonstrated similar expression levels in the epithelium of healthy colon tissue as compared with the normal tissue adjacent to the pT1 lesions (online supplemental figure 6). Similarly, the genes of interest that were deregulated in the stroma during CRC tumourigenesis demonstrated comparable expression between vimentin segments in normal areas next to pT1 lesions and vimentin-positive regions in the healthy colon (online supplemental figure 7). These results demonstrate that the genes identified by our approach became deregulated during early events of CRC tumourigenesis as their expression was largely similar between healthy colon and regions identified as ‘normal’ in our pT1 lesions.

10.1136/gutjnl-2022-327608.supp12 Supplementary data

10.1136/gutjnl-2022-327608.supp13 Supplementary data

In sum, our approach identified markers that not only inform on biological processes associated with malignant progression but, importantly, that can also be employed in a clinical context as biomarkers.

### Biological processes associated CRC onset are distinct between epithelium and stroma

**Figure 4:**
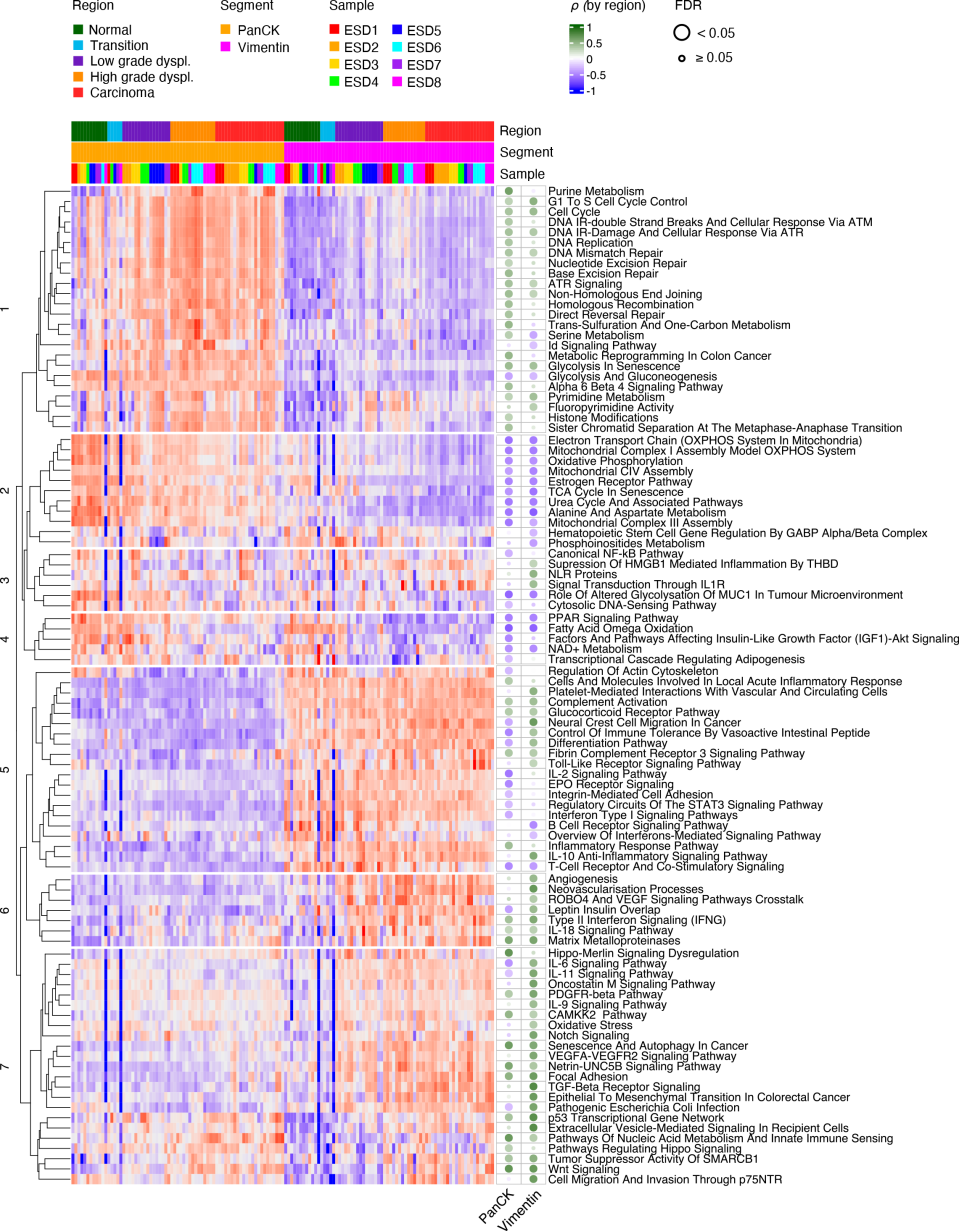
Key biological pathways associated with advancing histology in epithelial and stromal compartments. AOIs are ordered by segment, region and by sample ID. Unsupervised clustering of gene set enrichment scores (ssGSEA) calculated using gene sets from the WikiPathways database. All pathways with a significant association with histology, either in PanCK or Vimentin segments, are included (FDR<0.05). Main clusters of identified pathways are indicated with white horizontal lines. The corresponding Spearman correlation coefficient, Rho, between histology as ordinal variable and the enrichment scores are indicated for each pathway in PanCK and Vimentin segments separately (dotted heatmap). FDR is calculated using Benjamini-Hochberg method. An unsupervised version of this heatmap, using unsupervised clustering for both rows and columns, is included as online supplemental figure S8. AOI, area of illumination; FDR, false discovery rate; PanCK, pan-cytokeratin; ssGSEA, single sample gene set enrichment.

For a comprehensive assessment of alterations during progression from normal tissue to carcinoma, we performed pathway enrichment analysis with a collection retrieved from the WikiPathways database.^18^ Based on pathway enrichment scores, most cancer and high-grade dysplasia AOIs of the epithelial segment clustered together, demonstrating that these regions are clearly distinct from histologies corresponding to normal and transition regions as well as low-grade dysplasia (online supplemental figure 8). We identified seven distinct clusters of pathways (C1–C7) with different degrees of association with malignant transformation (figure 4, online supplemental figure 8). Three clusters (C1, C2 and C4) grouped pathways that were mainly active in the epithelial segment. C1-pathways included biological processes associated with proliferation and DNA damage repair, which consistently increased during CRC onset. In concordance with the higher proliferation demands^22^ the serine metabolism pathway was also increased from normal tissue to cancer. The majority of pathways clustered in C2 were associated with mitochondrial function, and demonstrated a decrease during malignant transformation in the epithelial fraction. A decreased enrichment of genes associated with oxidative phosphorylation and concurrent increase in enrichment score of the glycolysis pathway, points toward a metabolic switch to aerobic glycolysis.^23 24^ Similarly, pathways in C4 decreased during CRC onset, which mainly consisted of pathways related to lipid metabolism, including Fatty Acid Omega Oxidation and peroxisome proliferator-activated receptor (PPAR) signalling. PPARs are activated by fatty acids, and anti-neoplastic (eg, anti-proliferative and pro-apoptotic) effects of PPARγ have been described.^25^ These alterations in C4 were paralleled in the stroma, indicating their coordinated regulation in both biological compartments. Activity of pathways clustered in C3 were observed both in the epithelium and stroma, and demonstrated variable associations with tumour progression. These included Role of Altered glycosylation of MUC1 in the tumour microenvironment, Nod-like receptor (NLR) family proteins and cytosolic DNA sensing. The pathways in clusters C5, C6 and C7 were mainly associated to the stromal compartment. Pathways in C5 reflected mainly immune-related pathways that were clearly deregulated during tumourigenesis. The stroma of dysplastic and invasive carcinoma regions was particularly enriched for pathways in C6 and C7, including transforming growth factor (TGF)-β receptor signalling, focal adhesion, matrix metalloproteinases and neovascularisation, all associated with an increased malignant behaviour of tumours. Specific pathways in C7 were associated with high-grade dysplasia and carcinoma histologies, both in epithelial and stromal compartment. These included pathways typically associated with CRC progression such as Wnt and p53 signalling but also pathways that have been less explored in the context of this disease like innate immune sensing. These results demonstrate marked transcriptomic alterations during tumourigenesis and the existence of biological processes related to malignancy that are specifically enriched in the epithelial or stromal compartments of a tumour.

10.1136/gutjnl-2022-327608.supp14 Supplementary data

### Immune-related alterations during CRC progression

**Figure 5:**
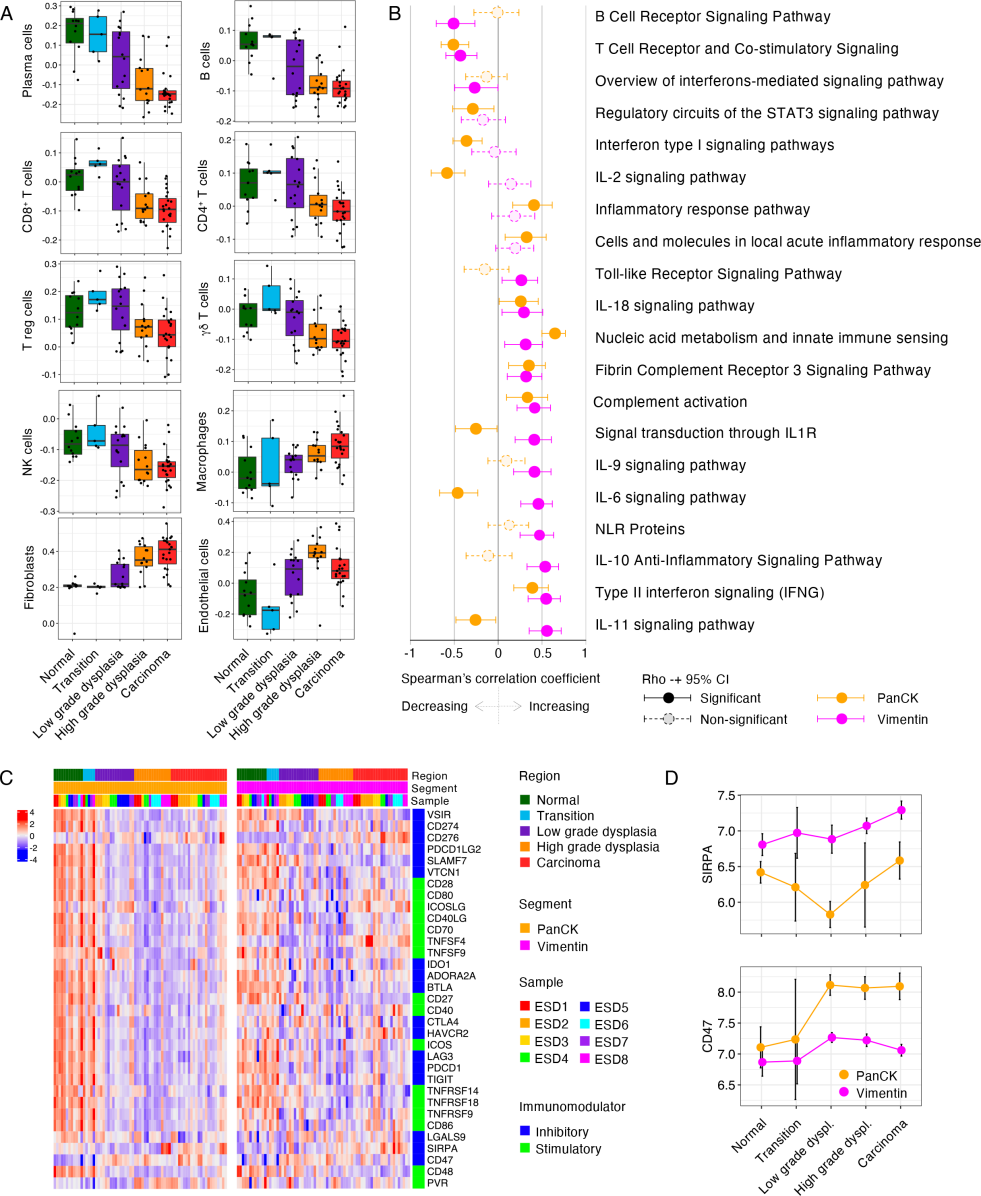
Immune-related alterations in relation to CRC histology. (A) Boxplots of deconvoluted abundancies of distinct immune cell populations across histologies in the Vimentin segment. (B) Forest plot of Spearman’s Rho and corresponding 95% CI for the correlation between enrichment score of immune-related pathways from WikiPathways and histology as ordinal variable. Correlation was assessed in PanCK and Vimentin segments separately. (C) Expression of immunomodulators across distinct regions in the Vimentin segment. AOIs (columns) are ordered by histology and subsequently by sample ID. Expression z-score was calculated in epithelial and stromal segments separately. Type of immunomodulator, either inhibitory or stimulatory, is indicated. An unsupervised version of the heatmap with PanCK and Vimentin regions combined, using unsupervised clustering for both rows and columns, is included as online supplemental figure 9. (D) Line graphs of expression of *CD47* and *SIRPA* during tumourigenesis. Mean enrichment score and corresponding 95% CI are indicated. CRC, colorectal cancer; log2FC, log2 fold change; PanCK, pan-cytokeratin; ROI, region of interest.

Intrigued by the observed changes in immune-related pathways during CRC onset, we subsequently focused on alterations to the tumour (immune) microenvironment between regions of distinct histology. First, we performed immune cell deconvolution to estimate the relative abundancies of specific cell subsets in each AOI. In the stromal compartment, we defined multiple immune cell subsets with different relative abundancies between histological regions (ANOVA; Benjamini-Hochberg method FDR<0.05). The relative abundance of plasma cells, B cells, CD8 T cells, CD4 T cells, T regulatory cells, γδ T cells and natural killer cells, decreased from normal tissue to carcinoma (figure 5A). On the other hand, the relative abundance of fibroblasts, endothelial cells and macrophages increased during tumourigenesis (figure 5A). These results point to the development of an immune-suppressed microenvironment accompanying tumour progression, which is also in line with the upregulation of immunosuppressive signalling pathways like TGF-β (figure 4C, Cluster C7).^26–28^

10.1136/gutjnl-2022-327608.supp15 Supplementary data

To define immune-related alterations beyond the estimation of the cellular composition of the tumour microenvironment, we re-evaluated the altered enrichment of WikiPathways focusing on the immune-related gene sets. The majority of immune-related signatures (20/27) were altered during malignant transformation, reflecting substantial changes in the immune-microenvironment (figure 5B). Consistent with the decrease in deconvoluted B cells abundance, the enrichment scores of ‘B Cell Receptor Signaling Pathway’ decreased from normal tissue to carcinoma. Other pathways that inversely correlated with advancing histology included ‘T Cell Receptor and Co-stimulatory Signaling’ and ‘Overview of interferons-mediated signaling pathway’, reflecting a reduced number of T cells in the microenvironment. Different cytokine-related pathways were upregulated in the stroma during the progression from normal mucosa to carcinoma, including interferon γ, interleukin (IL)-11, IL-10, IL-6, IL-9, IL1R and IL-18 signalling pathways. Many of these cytokines have been described in the context of innate immunity (IL-1, IL-6, IL-10 and IL-18)^29^ (figure 5B). We also identified two pathways linked to innate immunity that were consistently increased in both segments, including the ‘Fibrin Complement Receptor 3 Signaling Pathway’, ‘Nucleic acid metabolism and innate immune sensing’ and ‘NLR family proteins’. Interestingly, innate immune sensing genes were particularly increased in the epithelial compartment, suggesting a connection between the activation of this pathway in epithelial cells and the development of an innate immune response.^30^ Genes that contributed to the observed increase in this signature included *DDX58*, *OAS1* and *MAVS*, which are essential for antiviral innate immunity.^31^ NLR family proteins have been implicated in innate immune sensing of microbes and infection-associated physiological changes,^32^ and were specifically upregulated in the vimentin segment during tumour progression.

Next, we focused on the expression of immunomodulatory genes associated with cancer immunity. As expected, the majority of immunomodulatory gene expression was derived from stromal segments (online supplemental figure 9). A large number of immunomodulators were increased in the stroma of normal and transition tissues (figure 5C, online supplemental figure 9), which likely reflects the tight balance that is in place in colorectal tissues to provide immune defence but prevent exacerbated inflammation. Interestingly, a specific group of immunomodulatory genes revealed a biphasic pattern (cluster 4, figure 5C), with high expression in normal and transition tissue, decreased expression in dysplastic tissues and slight increase in the stroma of carcinoma tissues. The ‘rescued’ expression of these immunomodulatory genes in carcinoma tissues suggests the late adoption of mechanisms of T-cell regulation during invasion of cancer cells to adjacent tissues. The immune immunomodulators *CD276* (B7-H3) and *SIRPA* (SIRPα) demonstrated increased expression in the stroma of high-grade dysplasia and carcinoma regions. B7-H3 can lead to decreased anti-tumour activity by T cells^33 34^ and its expression is known to be derived from myeloid cells but also from cancer-associated fibroblasts,^34 35^ which is in line with their increased abundance during tumour progression. Similarly, SIRPα, or signal regulatory protein alpha, expression is mainly reported in myeloid cells^36^ which is in line with the observed increase in deconvoluted macrophages during tumour progression (figure 5A) and, interestingly, the expression of its binding partner on tumour cells, *CD47*, was increased in the epithelial compartment in dysplastic and carcinoma tissues (figure 5D). Binding of CD47 on tumour cells to SIRPα on macrophages leads to inhibition of macrophage-mediated phagocytosis.^37–40^ Our observations suggest that this ‘don’t eat me’-signal provided by tumour cells could constitute a mechanism of immune escape in early-stage CRCs.

### Shift in macrophage populations during progression from normal tissue to cancer

**Figure 6:**
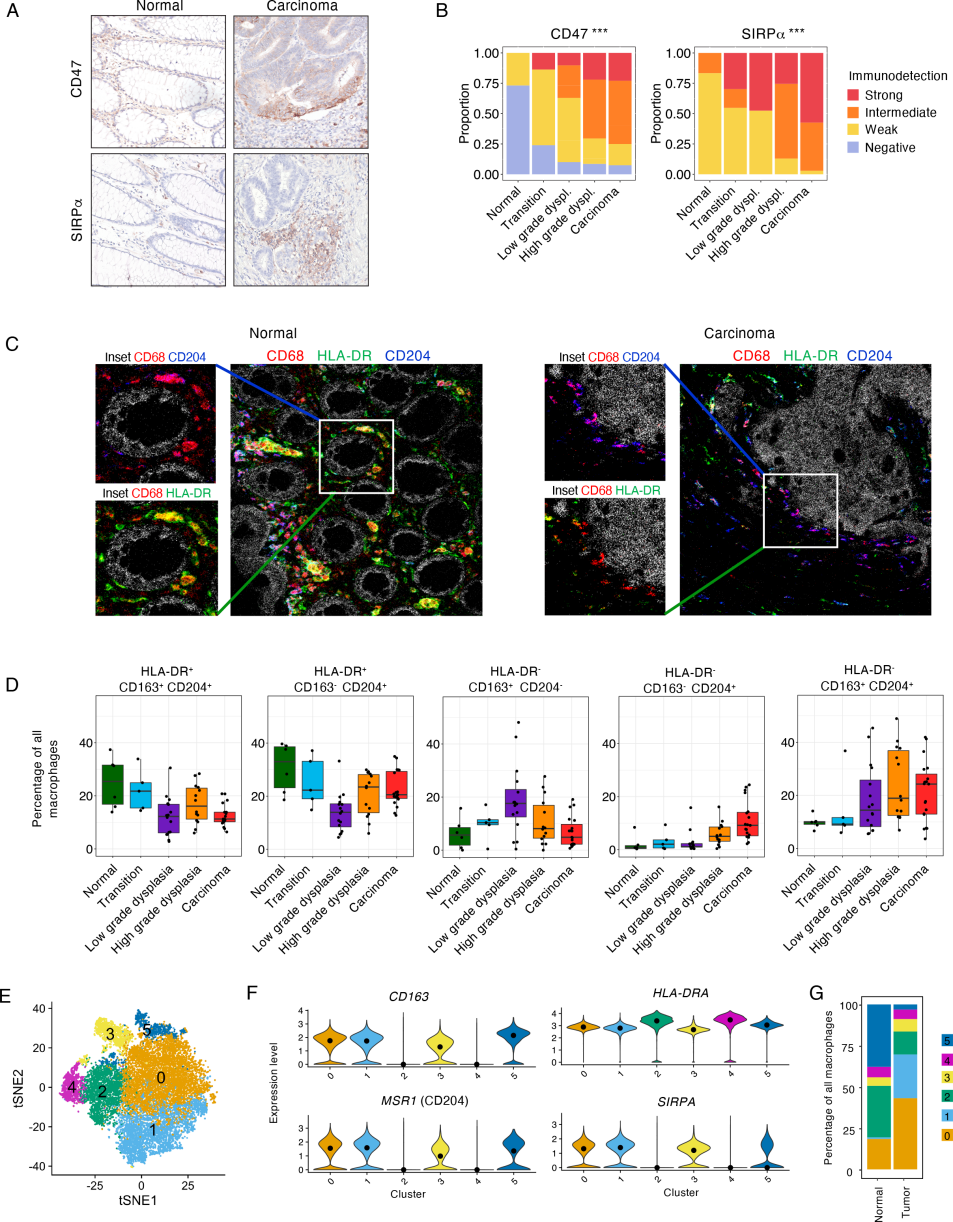
Upregulation of the CD47/SIRPα axis and corresponding shift in macrophage subpopulations during the stepwise progression from normal colorectal tissue to cancer. (A) IHC detection of SIRPα and CD47 in distinct histologies of pT1 CRC samples. (B) IHC scores for SIRPα and CD47 in the independent validation set of 18 pT1 CRC samples. Stacked barchart displays the distribution of samples according to the expression of SIRPα and CD47, per histological region. Significance of Spearman correlation is indicated by asterisks. ***p<0.001. (C) Visualisation of macrophage subpopulations by imaging mass cytometry in a normal region and carcinoma region of a pT1 CRC sample. Representative images showing HLA-DR^+^CD204^+^ macrophages in a normal region and HLA-DR^−^CD204^+^ macrophages in a carcinoma region. Myeloid markers: CD68 (*red*), CD204 (*blue*), and HLA-DR (*green*). The tumour is marked by ß-catenin (*white*). (D) Relative abundance of distinct macrophage subpopulations by region from normal colon tissue to carcinoma as defined by IMC. (E) tSNE embedding of all macrophages from 62 CRC samples (n=17 590 cells) and 35 adjacent normal samples (n=1257 cells) from the scRNA-seq data set by Pelka *et al*, 2021. Each dot represents a single cell. Six distinct clusters of macrophages were identified. (F) Violin plots showing the expression of *CD163*, *MSR1*, *HLA-DRA* and *SIRPA* in each of the identified macrophage clusters. (G) Prevalence of each macrophage cluster in normal tissue and cancer tissue. CRC, colorectal cancer; IHC, immunohistochemistry; IMC, imaging mass cytometry; scRNA-seq, single-cell RNA-sequencing; tSNE, t-Distributed Stochastic Neighbour Embedding

Intrigued by the potential upregulation of the CD47-SIRPα axis during early phases of CRC tumourigenesis, we investigated the protein expression of CD47 and SIRPα in the eight pT1 samples (online supplemental figure 10A) and 18 independent pT1 CRC tissues (figure 6A, B). We confirmed the expression of CD47 on epithelial cells and SIRPα on stromal cells. We validated the stepwise increase in the expression of these proteins from normal colorectal tissue to carcinoma (figure 6A, B) (CD47, Spearman Rho=0.47, p=6.5×10^−6^; SIRPα, Spearman Rho=0.60, p=2.2×10^−9^).

10.1136/gutjnl-2022-327608.supp16 Supplementary data

To better characterise and substantiate the alterations in the immune microenvironment during CRC tumourigenesis, we performed imaging mass cytometry (IMC) on consecutive sections derived from the same lesions. An increased density of dendritic cells, monocytes, granulocytes, innate lymphoid cells, fibroblasts and vessels was observed during the transition from normal tissue to cancer (online supplemental figure 10B). These densities were mostly in line with the estimates by deconvolution of the gene expression data (figure 5A) with some exceptions, including macrophages. Interestingly, while the density of macrophages did not deviate substantially from normal, through dysplastic to cancer regions, the relative abundance of specific macrophage populations changed substantially (figure 6A, B). Macrophage populations were identified by CD68 expression and delineated based on the expression of CD163, HLA-DR and/or CD204 expression. The relative frequency of HLA-DR^+^ macrophages (supposedly pro-inflammatory subsets) decreased during the stepwise progression from normal tissue to colon cancer (figure 6C, D). Conversely, the relative frequency of HLA-DR^−^CD204^+^ macrophage subpopulations, irrespective of CD163 expression, increased during CRC progression (figure 6C, D). HLA-DR^−^CD163^+^CD204^−^ macrophages demonstrated a biphasic pattern with an initial increase until areas of low-grade dysplasia and subsequent decrease from low-grade dysplasia to cancer (figure 6B). Based on these data, we speculated that the expression of SIRPα in pT1 CRCs was most likely derived from HLA-DR^−^CD204^+^ macrophage populations (figure 6B).

We investigated this hypothesis by interrogating the publicly available scRNA-seq data set from CRC and adjacent normal tissues by Pelka *et al*.^11^ This data set includes single cells derived from 62 CRC samples and 35 adjacent normal samples. We separately clustered all macrophages from this data set to derive six subpopulations (figure 6C, online supplemental figure 11). These macrophage subpopulations displayed specific gene expression profiles (online supplemental figure 11) and demonstrated distinct expression levels of *CD163*, *MSR1* (CD204), *HLA-DRA* and *SIRPA* (figure 6D, online supplemental figure 11). Macrophage populations expressing high levels of *SIRPA* (clusters 0, 1, 3), co-expressed *MSR1* (CD204) and displayed lower expression of *HLA-DRA* compared with other macrophage subsets (figure 6E, F). Consistent with our observations, we noted a clear increase in the prevalence of *SIRPA^+^MSR1^+^-*expressing macrophage subpopulations in tumour tissues (77.3% of all macrophages) compared with normal tissues (24.6% of all macrophages) (figure 6G). Altogether, these results provide evidence for a shift in macrophage populations during the stepwise progression from normal tissue to cancer, accompanied by the upregulation of the CD47/SIRPα axis.

10.1136/gutjnl-2022-327608.supp17 Supplementary data

## Discussion

Here, we combined multiregion transcriptomic profiling by DSP with high dimensional characterisation of cancer immune microenvironments using IMC to assess the biological changes during the onset of CRC. This approach allowed a unique and comprehensive assessment of alterations at early stages of CRC tumourigenesis which could not have been provided by bulk or single cell transcriptomic studies.

Beyond re-capitulating biological pathways described as hallmarks of cancer,^2^ our approach provided context to the relevant biological compartment to which these alterations occur. Hereby, we were able to define alterations in oncogenic, metabolic and immune-related processes during CRC tumourigenesis. In particular, in the stromal segment, we observed substantial changes in cellular composition from normal tissue to cancer. Consistent with observations in the immune composition in precancerous polyps,^13^ we noted a decrease in B cells and plasma cells and an increase in fibroblasts from normal to cancer regions in pT1 CRCs. Furthermore, IMC identified stepwise increases in densities of vessels, monocytes, dendritic cells and granulocytes. In parallel, we noted a shift in macrophage populations from HLA-DR^+^ subsets, which supposedly represent pro-inflammatory subsets, to immunosuppressive HLA-DR^−^CD204^+^ macrophage subpopulations during CRC progression.^41 42^

The influence of the adaptive immune response on CRC has been well established.^43 44^ Although a large proportion of the immune cells that infiltrate CRCs are of myeloid origin, our understanding of the contribution of the innate immune system on cancer progression is less advanced.^45^ Tumour-elicited inflammation is thought to be caused by the breakdown of the epithelial barrier during tumourigenesis that enables microbial products translocate from the intestinal lumen to activate tissue-resident myeloid cells.^46 47^ In addition, necrotic cell death as a consequence of hypoxic conditions and lack of sufficient nutrients has been described to induce innate and adaptive immune responses.^46^ In this study, we observed an increase in genes associated with innate immunity during the stepwise transition from normal tissue to carcinoma. Already in the transition areas from normal tissue to dysplasia, we detected an upregulation of transcripts for *CFB* and *CCL20* in the epithelium and *CXCL1* in the stroma. Furthermore, we identified an upregulation of major components of the complement pathway, and of cytokines related to innate immunity (IL-1, IL-6, IL-10, IL-18 and TGF-β).^29^ At pathway level, the upregulation of nucleic acid metabolism and innate immune sensing during tumour progression was evident in the epithelium of pT1 cancers. Furthermore, we found evidence for microbial sensing activity via NLR family members in the stromal compartment. Roles for both tumour-presenting and antigen-presenting cells have been described in mediation of nucleic acid sensing.^48^ A link between Nod1 and colorectal tumourigenesis has been described, in which the bacterial sensor Nod1 exerts immunosuppressive potential via arginase activity, thereby promoting tumourigenesis by creating a tumour-permissive microenvironment.^49^ These alterations are in line with chronic inflammation, one of the hallmarks of cancer,^50^ which can stimulate neoplastic transformation and sustain disease progression.

Our segmentation approach enabled us to define ligand and corresponding receptor relations of immunomodulator genes. We defined a simultaneous upregulation of *SIRPA* in the stroma and *CD47* in the epithelium during the stepwise progression of CRC. We found that HLA-DR^−^CD204^+^ macrophage populations that increase in frequency during CRC tumourigenesis are expressing *SIRPA*. As CD47 expression provides a ‘don’t eat me’-signal to macrophages expressing SIRPA,^38–40^ this could be an effective mechanism of immune escape in early-staged CRCs. Therefore, the CD47/SIRPA axis might also represent an interesting therapeutic candidate for these cancers. Immunotherapeutic strategies that block this innate immune checkpoint are currently in early-phase clinical trials.^38 51 52^

This work also provides a framework for discovery of novel biomarkers to aid early disease interception and clinical management. Population screening programmes aim at the early diagnosis and treatment of CRC, thereby reducing patient morbidity and mortality.^53^ These programmes have resulted in an increased number of patients diagnosed with early-stage CRC (eg, pT1 CRC)^54^ that are amenable to less invasive clinical interventions that include endoscopic removal of tumours. The dilemma whether conservative (eg, endoscopic) procedures should be followed by extensive curative procedures (eg, surgical resection) in pT1-staged CRCs, is generally addressed by morphological and histological evaluation of the lesions.^55 56^ However, this strategy is suboptimal, as reflected by substantial over-treatment of patients (~80–90%).^57 58^ In advanced stages of CRC, microsatellite instability status, consensus molecular subtype-classification and Immunoscore have been associated with clinical outcome.^4 7 59^ These parameters are unable to accurately predict clinical outcomes for pT1 CRCs,^60^ highlighting the need for novel markers by specifically investigating early-stage CRCs. Our approach identified novel potential biomarkers by the specific examination of dynamics in gene expression from normal tissue to cancer in pT1 CRCs. Future studies in larger cohorts of patients will be required to study the relation with clinical parameters and prognosis. Furthermore, the technology and approach could also be highly valuable to investigate potential biomarkers to identify individuals with increased risk of cancer by examination of samples from patients with CRC and healthy controls.

In conclusion, this study demonstrates the application of digital spatial profiling to early-stage CRC and provides insights in the biological processes that accompany the stepwise progression of CRC. Our results indicate an essential role for innate immunity in CRC onset. Furthermore, our approach provides a framework for identification of biomarkers that are consistently altered during CRC tumourigenesis. These insights pave the way to improving the clinical management of patients with early-stage CRC.

10.1136/gutjnl-2022-327608.supp5 Supplementary data

10.1136/gutjnl-2022-327608.supp6 Supplementary data

## Data Availability

Data are available in a public, open access repository. The GeoMx raw and normalised data is available via FigShare https://doi.org/10.6084/m9.figshare.16818415. Imaging Mass Cytometry files (mcd files) and the corresponding annotation file are shared on BioImage Archive (Accession number S-BIAD587, https://www.ebi.ac.uk/biostudies/bioimages/studies/S-BIAD587).
