# Supplementary figures and images for: Transcriptomic and immunophenotypic profiling reveals molecular and immunological hallmarks of colorectal cancer tumourigenesis
Source: Gut. 2022 Nov 28;72(7):1326–39. doi: 10.1136/gutjnl-2022-327608 (PMC10314051; doi:10.1136/gutjnl-2022-327608)

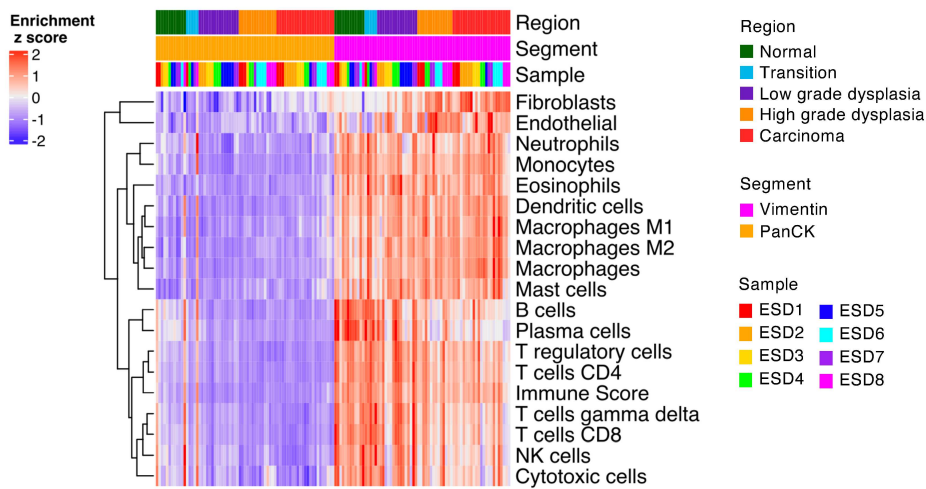

Supplement: Supplementary data [file gutjnl-2022-327608supp007.pdf]

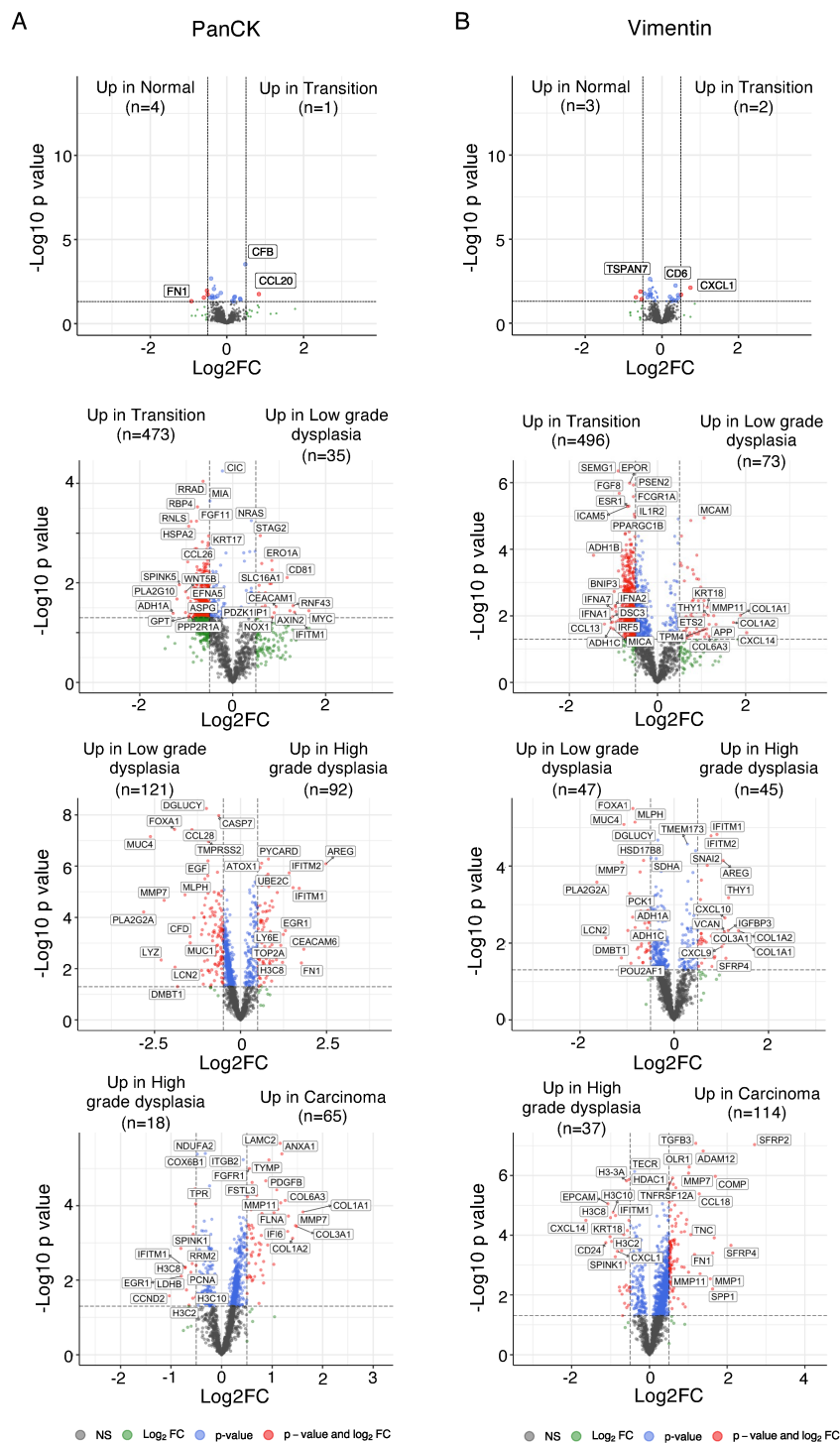

Supplement: Supplementary data [file gutjnl-2022-327608supp009.pdf]

Genes of interest from epithelial segment

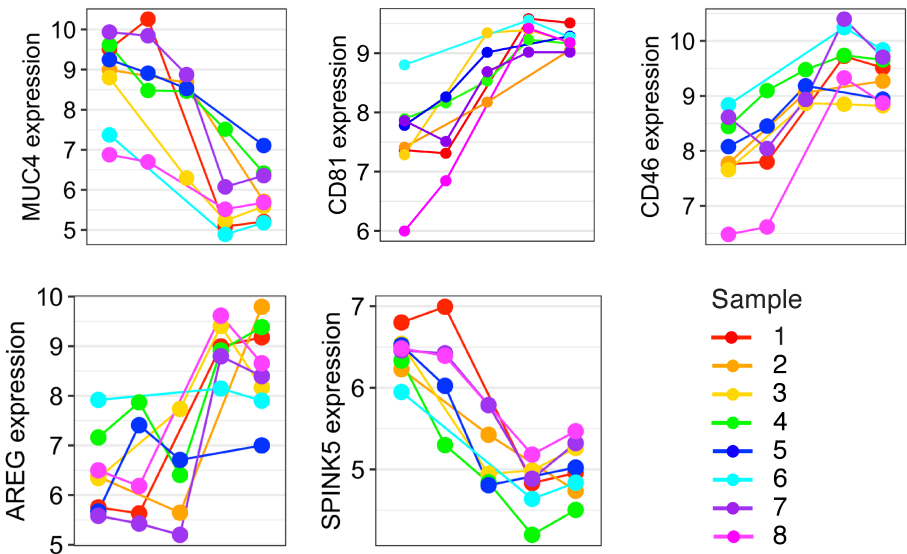

Genes of interest from stromal segment

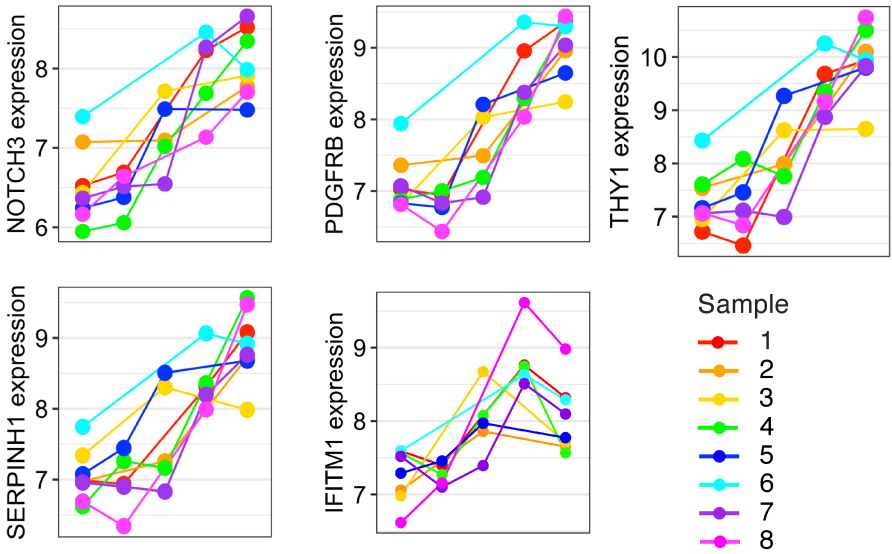

Supplement: Supplementary data [file gutjnl-2022-327608supp010.pdf]

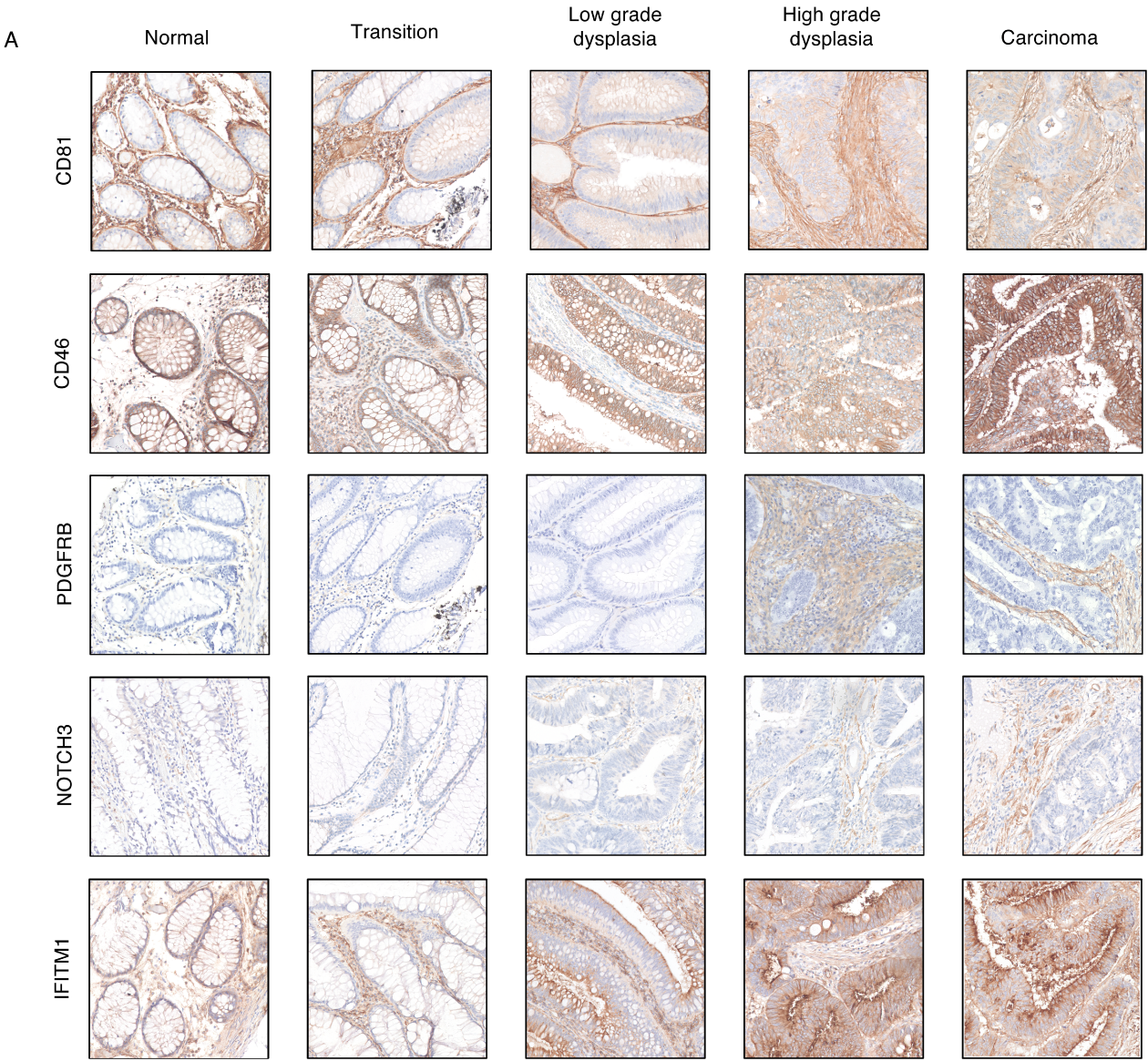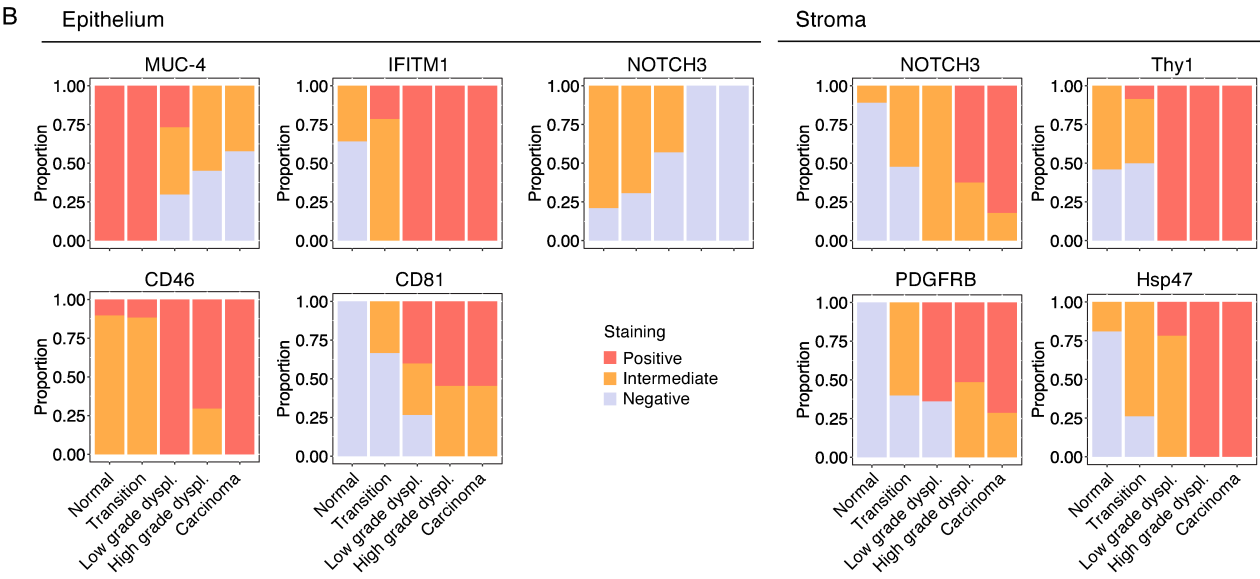

Supplement: Supplementary data [file gutjnl-2022-327608supp011.pdf]

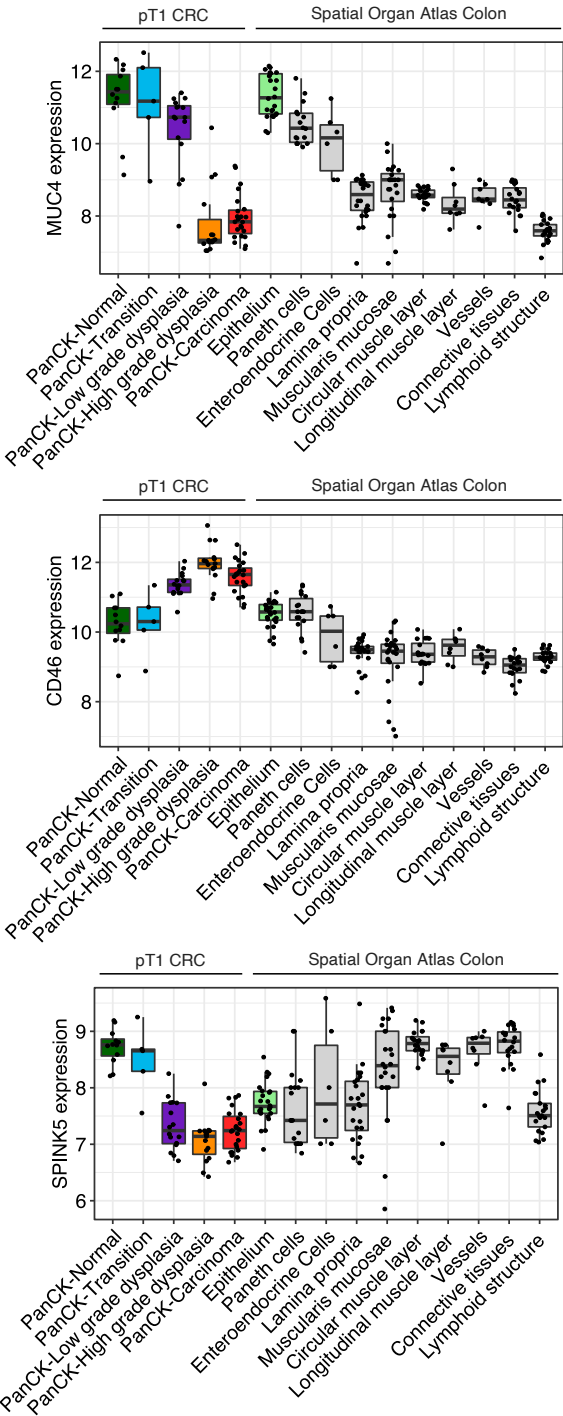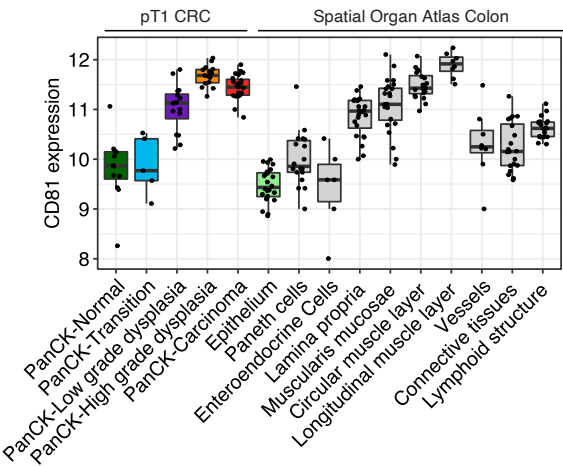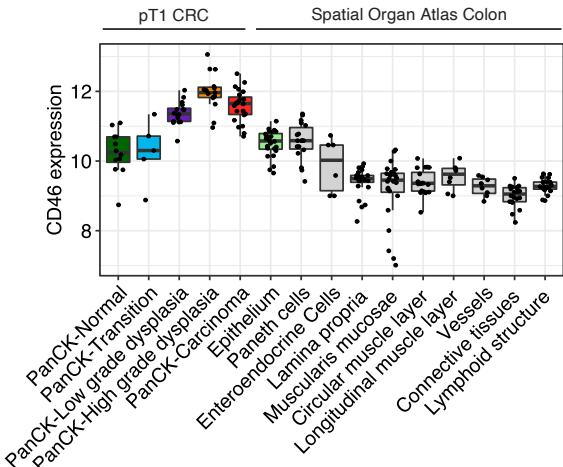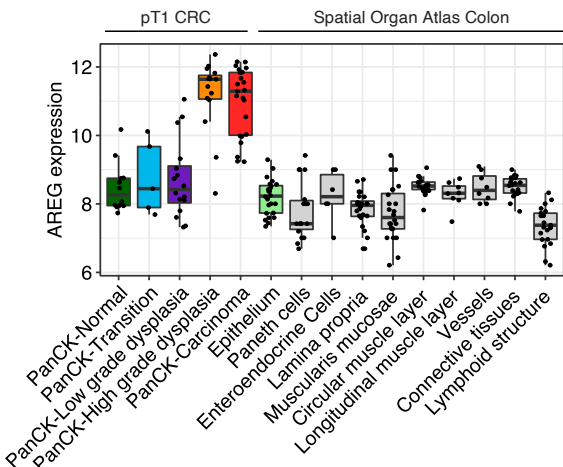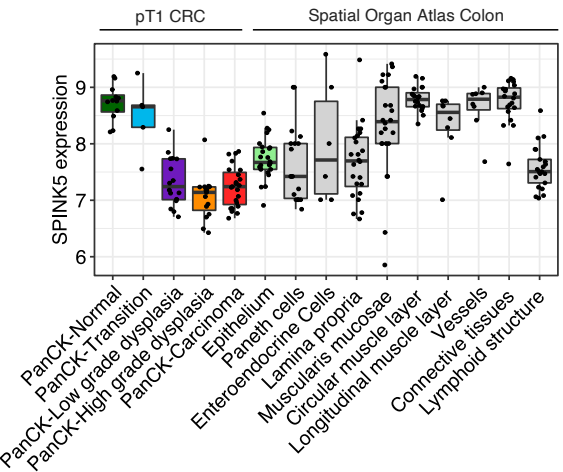

Supplement: Supplementary data [file gutjnl-2022-327608supp012.pdf]

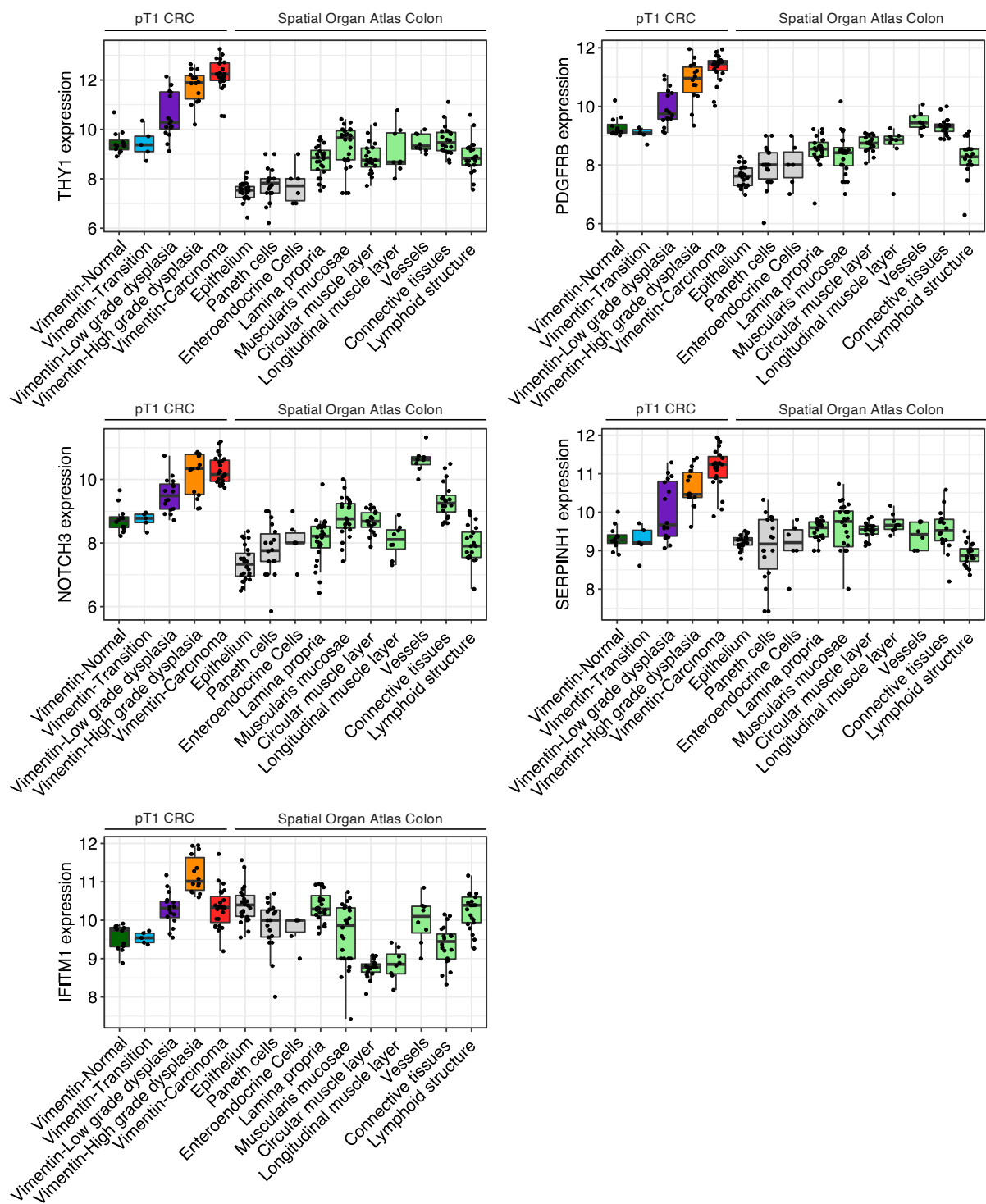

Supplement: Supplementary data [file gutjnl-2022-327608supp013.pdf]

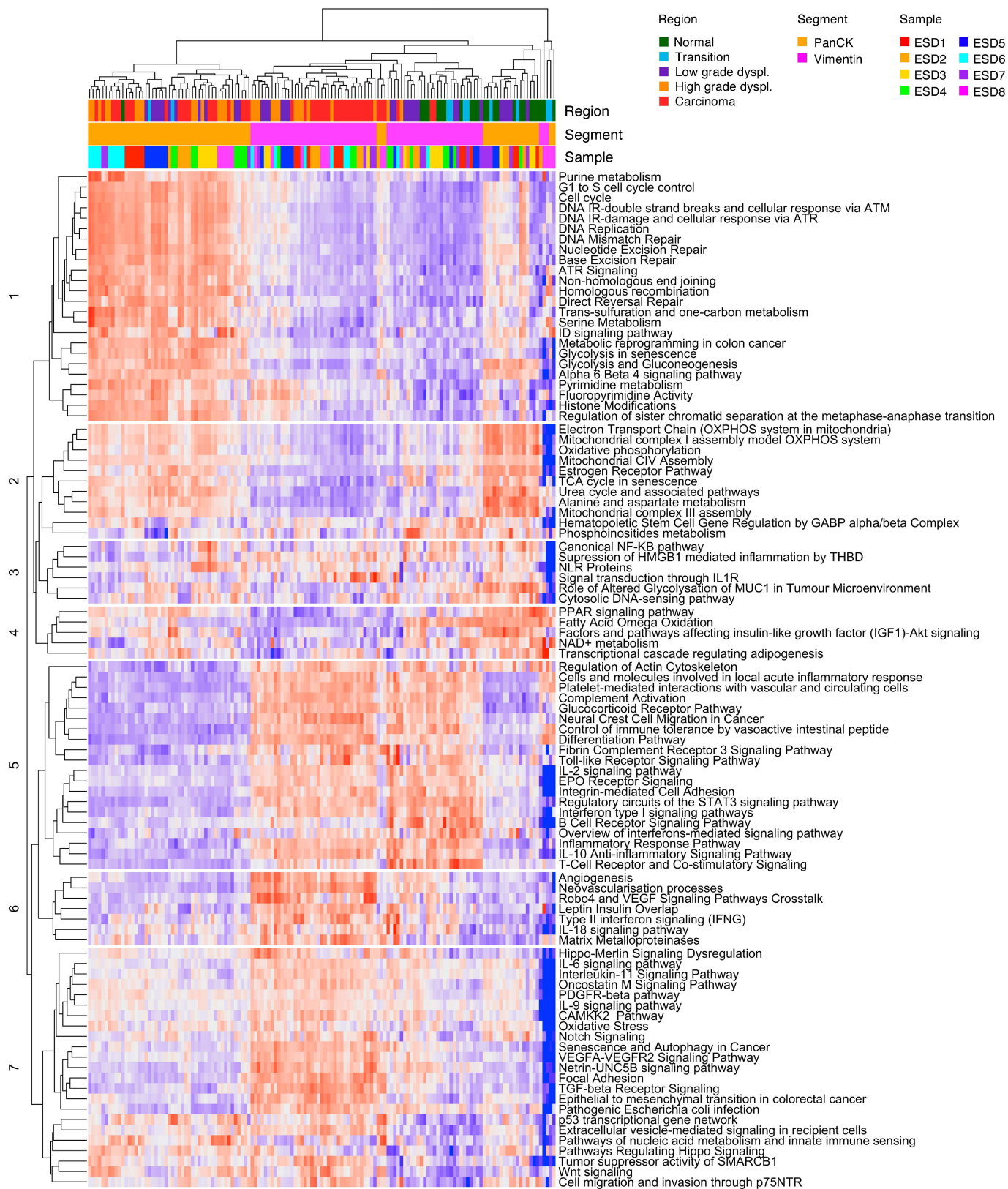

Supplement: Supplementary data [file gutjnl-2022-327608supp014.pdf]

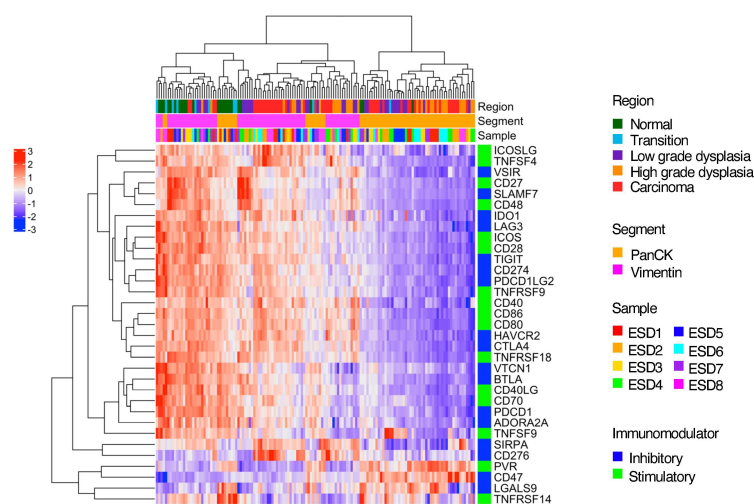

Supplement: Supplementary data [file gutjnl-2022-327608supp015.pdf]

A

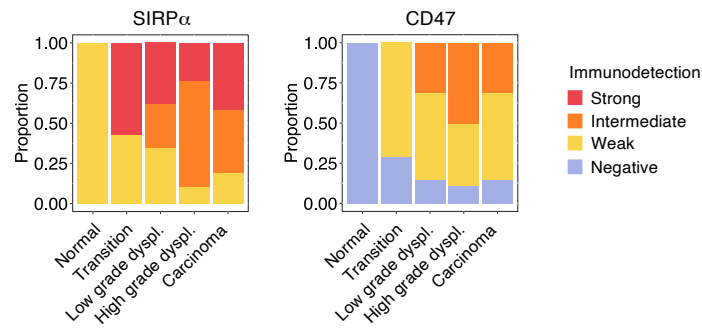

B

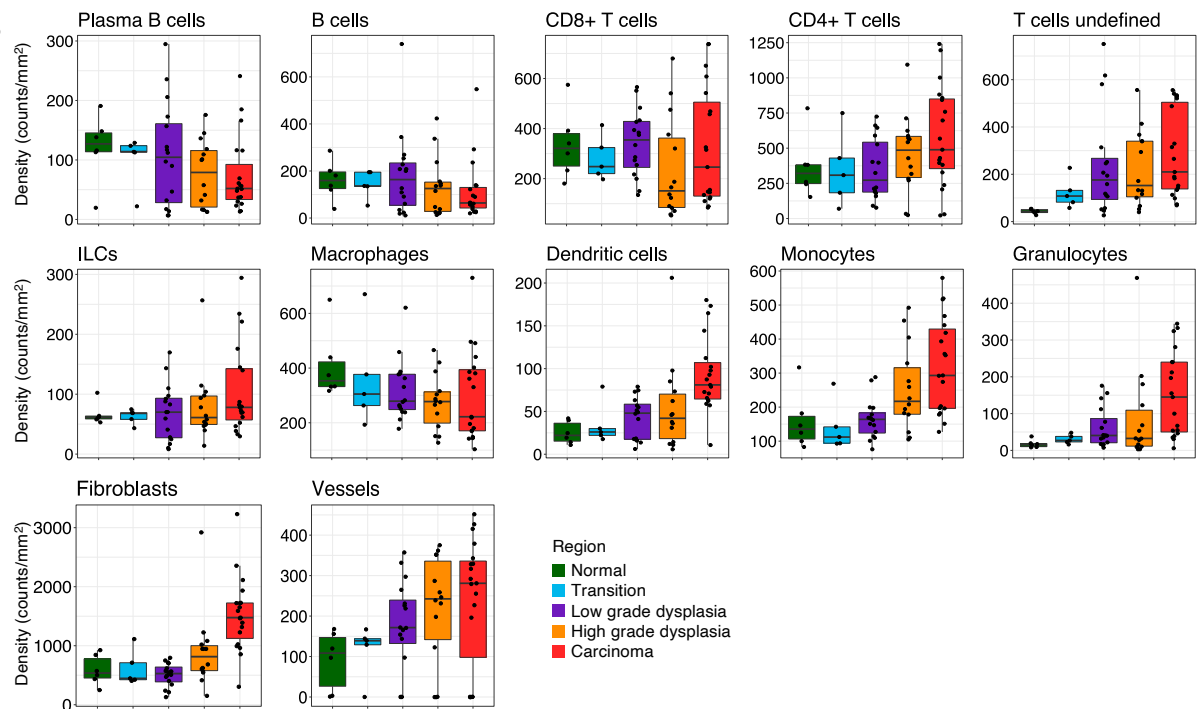

Supplement: Supplementary data [file gutjnl-2022-327608supp016.pdf]

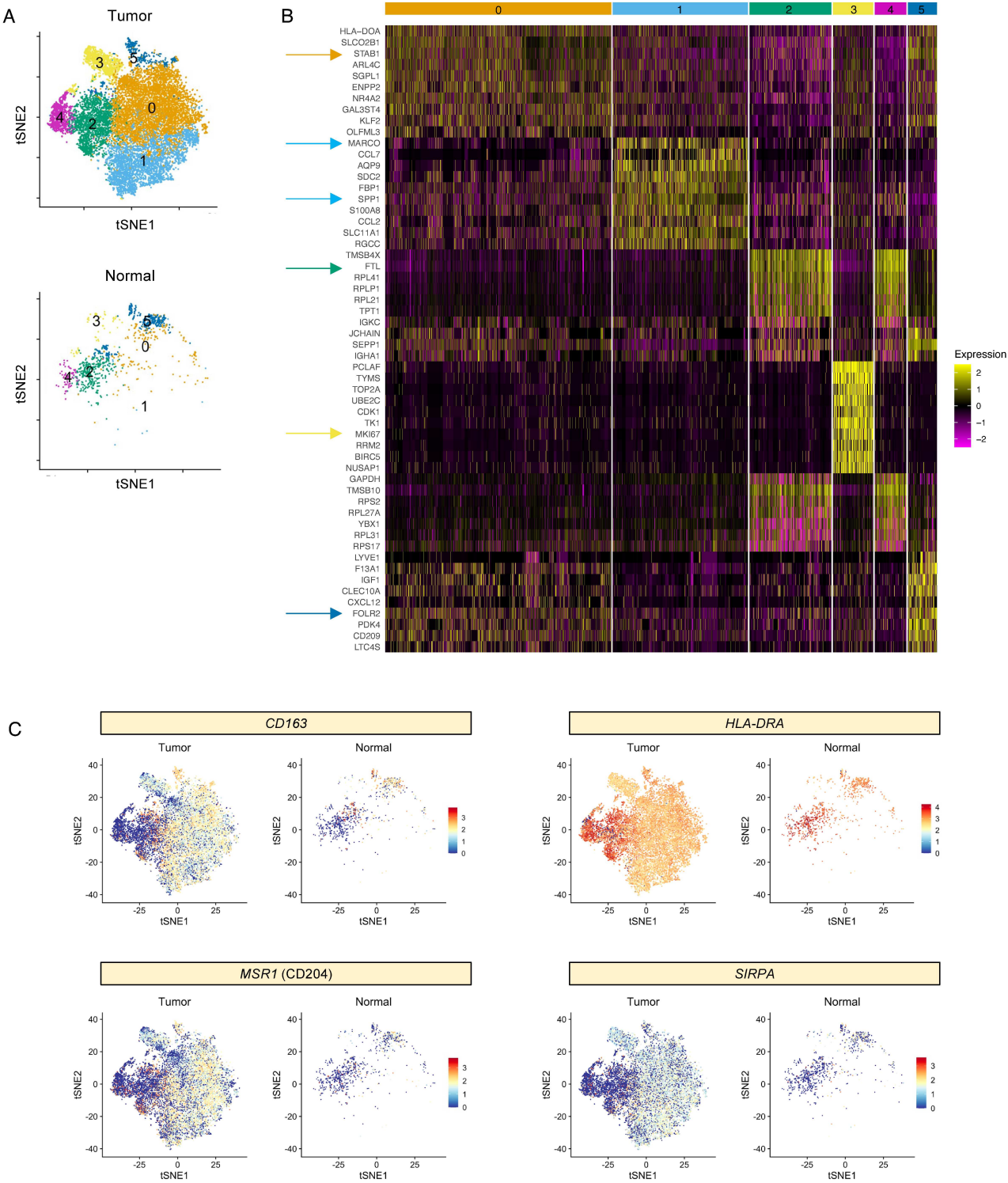

Supplement: Supplementary data [file gutjnl-2022-327608supp017.pdf]
