# Supplementary material for: Transcriptomic and immunophenotypic profiling reveals molecular and immunological hallmarks of colorectal cancer tumourigenesis
Source: Gut. 2022 Nov 28;72(7):1326–39. doi: 10.1136/gutjnl-2022-327608 (PMC10314051; doi:10.1136/gutjnl-2022-327608)
Supplement: Supplementary data [file gutjnl-2022-327608supp001.pdf]

Online Supplementary Methods

Immunohistochemistry

FFPE tissue sections of 4-μm thick were deparaffinized and rehydrated followed by heat-mediated antigen retrieval using either TE or Citrate buffer depending on primary antibody to be incubated (see Supplementary Table 1). Slides were blocked using SuperBlock (PBS) Blocking Buffer (Thermo Scientific, Cat# 37515) for 30 minutes at RT. Antibodies were diluted in PBS/BSA in predetermined optimal dilutions (see **Supplementary Table S1**). Tissue sections were incubated overnight at 4°C. Subsequently, slides were incubated with poly-HRP (Thermo Scientific, Cat# 21140) for 60 minutes and developed with DAB chromogen (1:50, DAKO #K3468). Sections were counterstained with hematoxylin (DiaPath #C0305). Slides were dehydrated and mounted with Micromount (Leica #3801731). Consecutive slides were used for the different antibodies. Stained slides were assessed by a pathologist. A simplified semi-quantitative IHC scoring was applied to all markers corresponding to Figure 2 of the paper, distinguishing completely negative (0), intermediate (1), including weak and/or heterogeneous staining, and positive (2), for homogeneous, positive staining.

Imaging Mass Cytometry

Antibody conjugation and immunodetection were performed as described in detail by Ijsselsteijn *et al*[1]. A complete list of antibodies used for immunophenotyping of the current cohort is provided in **Supplementary Table S2**. Consecutive sections of 4 μm were cut from the same ESDs as those profiled using GeoMx-DSP. First, FFPE tissue sections were incubated with anti-CD4 and anti-TCRdelta overnight at RT, which were subsequently detected using metal-conjugated secondary antibodies (goat anti-rabbit IgG and goat anti-mouse IgG, respectively; Abcam). Second, sections were incubated with 20 antibodies for five hours at RT. Third, sections were incubated overnight at 4°C with the remaining 19 antibodies. Fourth, sections were incubated with 0.125 μM Cell-ID intercalator-Ir (Fluidigm) to detect the DNA, and stored dry until measurement. For each sample, 1000x1000μm regions were selected based on the selected regions of interest in our spatial transcriptomics experiment and ablated using the Hyperion Imaging system (Fluidigm).

IMC data was normalized using semi-automated background removal in ilastik[2] as described previously[3] to control for variations in signal-to-noise between FFPE sections. The phenotype data was normalized at pixel level. Cell segmentation masks were created for all cells in ilastik and CellProfiler[4]. In ImaCytE , cell segmentation masks and normalized images were combined to generate single-cell FCS files containing the relative frequency of positive pixels for each marker per cell. Cells forming visual neighbourhoods in a t-distributed Stochastic Neighbour Embedding (t-SNE) in Cytosplore[6] were grouped and exported as

separate FCS files. The resulting subsets were imported back into ImaCyte and visualized on the segmentation masks. Major immune lineages were identified at the overview level of a hierarchical stochastic neigbour embedding (HSNE) analysis with 5000 iterations and as number of scales 2 in CytSplore[6]. Cells forming visual neighbourhoods in the H-SNE embedding were grouped into epithelial cells, lymphocytes, myeloid cells and stromal cells at sigma 8. Clusters were exported as separate FCS files, imported back into ImaCyte and visualized on the segmentation masks to confirm phenotypes. Similar cluster were further grouped in ImaCytE. Different subsets of macrophages were identified based on the expression of CD163, CD204, and HLA-DR. Plots were generated in R (v4.0.3 or later) using ggplot2 (v 3.3.5).

Single-cell RNA-sequencing analysis

We downloaded the publicly available scRNA-seq CRC dataset of Pelka *et al*.[7] using the Single Cell Portal (https://portals.broadinstitute.org/crc-immune-hubs/). This dataset consists of 62 CRC samples, and adjacent healthy tissue (n=36). Briefly, all macrophages (cluster MidWay “Macro”) were extracted for all samples. Initial re-clustering using the cells of this cluster revealed presence of cells that were of different lineage than macrophages (n= 1,556) which were therefore excluded. Re-clustering of all macrophages (n = 18,847) was performed with resolution parameter 0.3 using Seurat (v4.0.3). Non-linear dimensional reduction with tSNE was used to visualize the clustered cells split by variable SpecimenType (tumor or normal). A DotPlot was generated to identify clusters that were expressing *MSR1* (gene corresponding to CD204 protein) and *SIRPA*. To compare *CD47* expression between epithelial cells from tumor and adjacent normal samples in this dataset, the Broad Single Cell Portal was used (https://singlecell.broadinstitute.org/single_cell/study/SCP1162/human-colon-cancer-atlas-c295). All epithelial cells were included (i.e., no subsampling) and distribution plots were generated. Summary statistics were exported and data was re-plotted using ggplot in R.

References

1. Ijsselsteijn, M. E., van der Breggen, R., Farina Sarasqueta, A., Koning, F. & de Miranda, N. F. C. C. A 40-Marker Panel for High Dimensional Characterization of Cancer Immune Microenvironments by Imaging Mass Cytometry. *Front. Immunol.* **10**, (2019).
2. Berg, S. *et al.* ilastik: interactive machine learning for (bio)image analysis. *Nat. Methods* **16**, 1226–1232 (2019).

3. Ijsselsteijn, M. E., Somarakis, A., Lelieveldt, B. P. F., Höllt, T. & de Miranda, N. F. C. C. Semi-automated background removal limits data loss and normalizes imaging mass cytometry data. *Cytom. Part J. Int. Soc. Anal. Cytol.* (2021) doi:10.1002/cyto.a.24480.
4. Carpenter, A. E. *et al.* CellProfiler: image analysis software for identifying and quantifying cell phenotypes. *Genome Biol.* **7**, R100 (2006).
5. Somarakis, A., Van Unen, V., Koning, F., Lelieveldt, B. & Hollt, T. ImaCytE: Visual Exploration of Cellular Micro-Environments for Imaging Mass Cytometry Data. *IEEE Trans. Vis. Comput. Graph.* **27**, 98–110 (2021).
6. Höllt, T. *et al.* Cytosplore: Interactive Immune Cell Phenotyping for Large Single-Cell Datasets. *Comput. Graph. Forum* **35**, 171–180 (2016).

[dataset] 7. Pelka, K. *et al.* Spatially organized multicellular immune hubs in human colorectal cancer. *Cell* **184**, 4734-4752.e20 (2021).
