# Supplementary material for: Transcriptomic and immunophenotypic profiling reveals molecular and immunological hallmarks of colorectal cancer tumourigenesis
Source: Gut. 2022 Nov 28;72(7):1326–39. doi: 10.1136/gutjnl-2022-327608 (PMC10314051; doi:10.1136/gutjnl-2022-327608)

PanCK segment

Pearson correlation matrix of genes that positively associate with CRC onset (n= 242, mean correlation = 0.33)

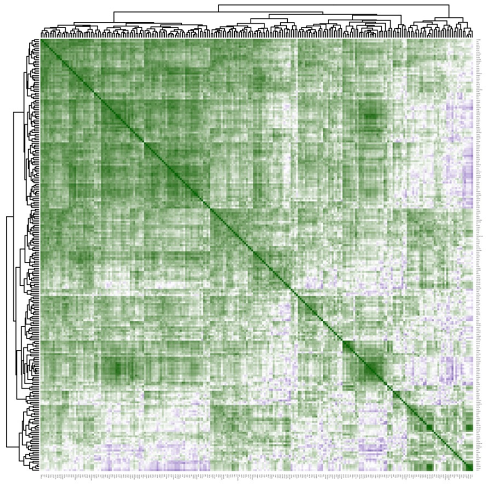

Pearson correlation matrix of genes that negatively associate with CRC onset (n= 1152, mean correlation = 0.64)

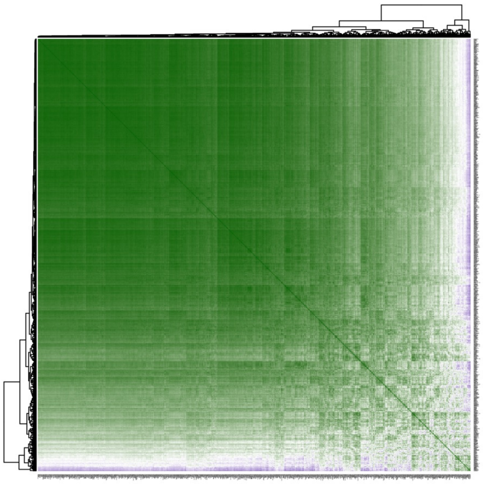

Vimentin segment

Pearson correlation matrix of genes that positively associate with CRC onset (n= 288, mean correlation = 0.36)

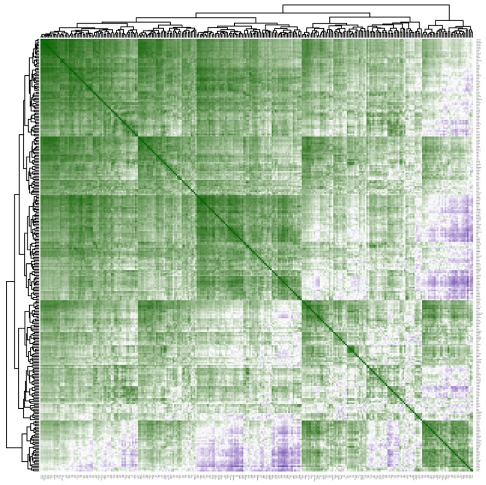

Pearson correlation matrix of genes that negatively associate with CRC onset (n= 857, mean correlation = 0.56)

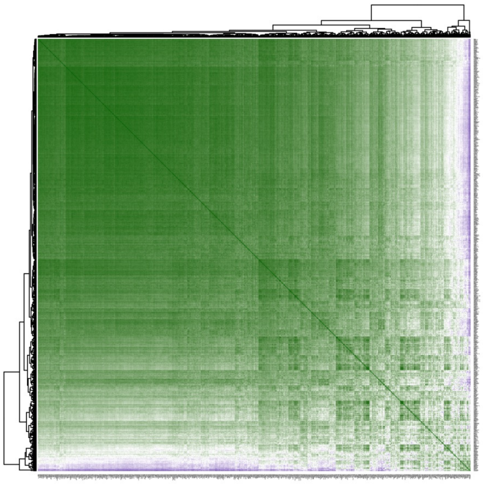

Supplement: Supplementary data [file gutjnl-2022-327608supp008.pdf]
